# Single‐Nucleus RNA Sequencing Reveals That Gabra6^+^ Neurons in Prefrontal Cortex Promote the Progression of PTSD After Shockwave‐Induced TBI

**DOI:** 10.1002/advs.202407000

**Published:** 2024-12-12

**Authors:** Xiaowei Fei, Zehan Zhang, Ya‐nan Dou, Weihao Lv, Hongqing Chen, Li Wang, Xin He, Wangshu Chao, Peng Luo, Zhou Fei

**Affiliations:** ^1^ Department of Neurosurgery Xijing Hospital Air Force Military Medical University Xi'an Shaanxi 710032 China

**Keywords:** cAMP signaling, Gabra6, PTSD, single‐nucleus RNA sequencing, TBI

## Abstract

Shockwave‐induced traumatic brain injury (TBI) results in the onset of post‐traumatic stress disorder (PTSD), triggered either by the TBI itself or other stressors. However, the interplay and underlying mechanisms of how these factors synergistically induce PTSD remain inadequately elucidated. Here, mice in the TBI (induced by biological shock tube blast injury) and PTSD (induced by single prolonged stress method) groups both displayed symptoms of PTSD behaviors, with the TBI+PTSD (composite model) group exhibiting more severe manifestations. The result of snRNA‐seq demonstrated a noticeable increase in the population of Gabra6^+^ neurons in the prefrontal cortex region of mice in the TBI+PTSD group. Knocking down cortical Gabra6 mitigated PTSD‐related behavioral outcomes. Mechanistically, the Smad3/4 complex activation led to the upregulation of Gabra6 expression in cortical neurons. Interaction of Gabra6 with Homer1 activated downstream cAMP signaling pathways. Homer1^KO‐Nestin^ mice show reduced susceptibility to PTSD. Subsequently, the efficacy of monoclonal antibody intervention at the 218 site of Gabra6 in ameliorating PTSD development is verified. This study suggests that TBI and stressors act as independent components in PTSD development, with Gabra6^+^ neurons pivotal in synergistically facilitating PTSD formation. Strategies geared toward minimizing exposure to singular or combined stressors may effectively diminish the risk of developing PTSD.

## Graphical abstract

This study suggests that traumatic brain injury and stressors act as independent components in post‐traumatic stress disorder (PTSD) development, with Gabra6^+^ neurons pivotal in synergistically facilitating PTSD formation. Smad3/4 complex activation led to upregulation of Gabra6 expression in cortical neurons. Interaction of Gabra6 with Homer1 activated cAMP signaling and intervention at 218th Glu of Gabra6 sequence effectively improved PTSD progression.

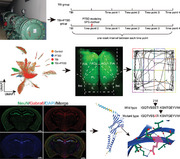

## 1 Introduction

Post‐Traumatic Stress Disorder (PTSD) denotes a severe mental health condition that emerges in individuals after intense psychological trauma.^[^ ^1^ ^]^ Triggered by significant catastrophic events, individuals may involuntarily become ensnared in recollections, manifested through palpitations, insomnia, heightened irritability, selective amnesia, diminished hope, and confidence in the future, and additional physiological and cognitive responses. Those who survive disasters like violent incidents, abuse, natural calamities, warfare, and terrorist assaults frequently endure profound psychological distress, rendering them predisposed to PTSD development.^[^ ^2^, ^3^, ^4^ ^]^ At now days, the extensive deployment of high‐explosive armaments is notably prevalent in both instances of terrorist and war attacks, which may generate shockwaves leading to various levels of traumatic brain injury (TBI) and substantial escalation of the probability of PTSD incidence.^[^ ^5^, ^6^ ^]^ As reported by Kontos et al., out of 22203 military personnel involved in the Iraq and Afghanistan conflicts, 2813 individuals (12.67%) sustained shockwave‐induced TBI, with 1476 individuals (6.64%) displaying clinical PTSD symptoms.^[^ ^7^ ^]^ A direct correlation exists between the dose of the shockwave (intensity of impact) and the severity of the reaction (clinical manifestations). Presently, predominant animal modeling methods employed in PTSD research revolve around the Single Prolonged Stress (SPS) paradigm.^[^ ^8^, ^9^, ^10^ ^]^ While some researchers leverage a controlled cortical impact apparatus for constructing TBI models to study PTSD.^[^ ^11^ ^]^ However, the precise establishment and interrogation of shockwave‐induced TBI models and their contributory role in PTSD's etiology remain largely uncharted territory.

Contemporary inquiries into the mechanistic underpinnings of PTSD primarily pivot on neural circuit modulation.^[^ ^12^ ^]^ Investigations have underscored the efficacy of high‐level thalamic regulation in ameliorating excessive defensive responses observed in PTSD.^[^ ^13^ ^]^ Notably, causal associations have been delineated between hyperactivation within the mediodorsal thalamic nucleus‐anterior cingulate cortex circuit and compromised fear extinction.^[^ ^14^, ^15^ ^]^ However, ambiguities persist regarding the nexus between fear conditioning, anxiety behaviors induced by shockwave‐induced TBI, and the involvement of the cortex. In addition, the cortex is a region of intricate cellular heterogeneity housing excitatory and inhibitory neurons alongside diverse non‐neuronal cell types.^[^ ^16^, ^17^ ^]^ There is intricately linked with alterations in neuronal structure and function correlating with changes in neuronal transcription. Conventional experimental approaches such as bulk RNA sequencing^[^ ^18^ ^]^ prove inadequate in deciphering cell‐specific transcription, impeding the identification of differentially expressed genes (DEGs) within the post‐shockwave‐induced TBI prefrontal cortex. To surmount this technical impediment, single‐nucleus RNA sequencing (snRNA‐seq) was employed to scrutinize transcriptomic alterations ensuing shockwave‐induced TBI in the prefrontal cortex.

Here, we pioneered a combined model amalgamating shockwaves with the SPS method to harmonize animal models with the clinical landscape of PTSD. Furthermore, leveraging glucocorticoids in conjunction with cell traction techniques, we engineered a cell model to address prevailing gaps in existing in vitro shockwave‐induced PTSD models. Subsequently, discerning cellular variances of prefrontal cortex between PTSD and the shockwave‐induced TBI‐PTSD concomitant groups, along with the analysis of DEGs, was undertaken to unveil alterations in prefrontal cortex cell gene expression post‐shockwave‐induced TBI and their implications in PTSD pathogenesis.

## 2 Result

### 2.1 TBI Induced by 4.5 MPa Shock Wave Developed PTSD

**Figure 1:**
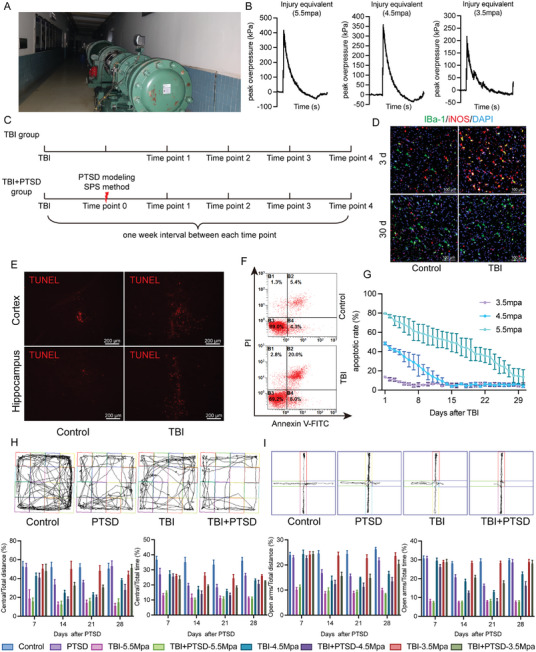
TBI‐induced PTSD mouse model construction and pathological damage detection. A) The photo of the BST‐I type biological shock tube. B) Overpressure peak pressures of different shock wave intensities. C) Diagram of group modeling. D) Cortical microglia of mice exposed to the 4.5 MPa shockwave at different time points and the red/green arrows represent M1/M2 microglia. E) Tunel staining of cortical and hippocampal cells at 3 days after TBI. F) Flow cytometry analysis of apoptosis levels in whole brain cells at 3 days after TBI. G) Quantitative results of flow cytometric analysis of apoptosis levels in the whole brain at each time point, (F_Interaction_(58, 180) = 10.80, *P < 0.0001*). The open field test (H) and elevated plus maze test (I) were used to detect the depression and anxiety levels of mice in each group at the 2‐week time point after modeling. H: Representative open field test behavioral trajectory map of mice exposed to a 4.5 MPa shock wave intensity at time point 2. Central/Total distance: F_Interaction_(21, 128) = 8.498, *P < 0.0001*; Central/Total time: F_Interaction_(21, 128) = 7.490, *P < 0.0001*; I: Representative elevated plus maze test behavioral trajectory map of mice exposed to a 4.5 MPa shock wave intensity at time point 2. Open arms/Total distance: F_Interaction_(21, 128) = 23.34, *P < 0.0001*; Open arms/Total time: F_Interaction_(21, 128) = 54.21, *p < 0.0001*. The data were analyzed using two‐way ANOVA (G, H, and I), and all data are expressed as the mean ± standard deviation. ^***^p < *0.001* and ^****^p < *0.0001* represent a statistically significant difference between the two groups. Each experiment was repeated three times.

The BST‐I type biological shock tube was utilized to create a shockwave‐induced TBI model (**Figure** **1**A), with controlled overpressure peak pressures of 3.5 MPa, 4.5 MPa, and 5.5 MPa to simulate TBI models of varying degrees of injury (Figure 1B). According to the experimental design, each group of mice was treated at different time points, and corresponding behavioral and pathological indicators were detected (Figure 1C). To determine the appropriate shockwave intensity, pathological examinations were performed on mice from different shockwave intensity groups. It was observed that the cortical microglia of mice exposed to the 4.5 MPa shockwave were activated at 3 days after TBI and returned to normal by day 30 (Figure 1D). TUNEL assay results revealed an increase in apoptosis of cortical and hippocampal cells at 3 days after TBI (Figure 1E). Two weeks after TBI, cortical neuronal damages were still significantly observed, rather than those of hippocampal neurons (Figure S1A,B, Supporting Information). Additionally, flow cytometric analysis of whole‐brain single‐cell suspensions also indicated an increase in cell apoptosis at 3 days after a 4.5 MPa shockwave (Figure 1F). The apoptosis rate in the 4.5 MPa group mice returned to normal around day 15, whereas a lower rate of apoptosis in the 3.5 MPa group, and a sustained level of apoptosis in the 5.5 MPa group were observed until 4 weeks after TBI (Figure 1G).

Behavioral results showed that mice in the PTSD group exhibited low exploratory behavior 2–3 weeks after modeling using the SPS method. Over time, the behavioral symptoms in mice gradually recovered after one month. This aligns with many studies that report a natural recovery (extinction of fear conditioning) process after PTSD modeling.^[^ ^19^, ^20^ ^]^ In groups with different intensities of TBI and TBI+PTSD, the behavior of mice in the 3.5 MPa shock wave group was similar to the control group at each time point. In contrast, mice exposed to a 5.5 MPa shock wave developed significant depression‐like behaviors as early as 7 days post‐modeling, and these symptoms did not recover after one month, showing no process of extinction of fear conditioning. Mice exposed to a 4.5 MPa shock wave exhibited significant behavioral inhibition from 1 to 2 weeks post‐modeling. Although their behavior gradually recovered after one month, in the TBI+PTSD composite model group, compared to the TBI‐4.5 MPa group, TBI hindered the normal recovery of behavioral function following PTSD. This indicates that 4.5 MPa TBI is an important factor exacerbating PTSD behavioral changes (Figure 1H,I; Table S1, Supporting Information). Thus, we opted for a 4.5 MPa shockwave to induce TBI as a causative factor for PTSD, to explore the impact of shockwave‐induced TBI on the development of PTSD. In addition, the Nissl staining results showed that the degree of neuronal damage in mice undergoing 4.5 MPa TBI was significantly higher than that in mice with a single PTSD group (Figure S1C, Supporting Information).

In in vitro experiments, TBI and PTSD were modeled using primary cortical neurons from mice. The findings indicated that Dex and Hcort had no significant impact on neuronal cell death, whereas the TBI group exhibited a significant increase in neuronal apoptosis rates (Figure S1D, Supporting Information). In addition, qPCR analysis showed an upregulation in the expression of inflammatory markers like IL‐1β, TNF‐α, and the depression‐associated gene SLC6A4 after hormonal treatment and TBI exposure. At the same time, there was a downregulation in the expression of IL‐10, ERCC6L2, and GAD‐1 (Figure S1E, Supporting Information). These data suggest that TBI induced by the 4.5 MPa shock wave promotes the development of PTSD in mice, and the symptoms of PTSD are further exacerbated when TBI is combined with SPS modeling.

### 2.2 Gabra6^+^ Neurons Accounted for an Increased Proportion of PTSD Mice after TBI as Revealed by snRNA‐seq

**Figure 2:**
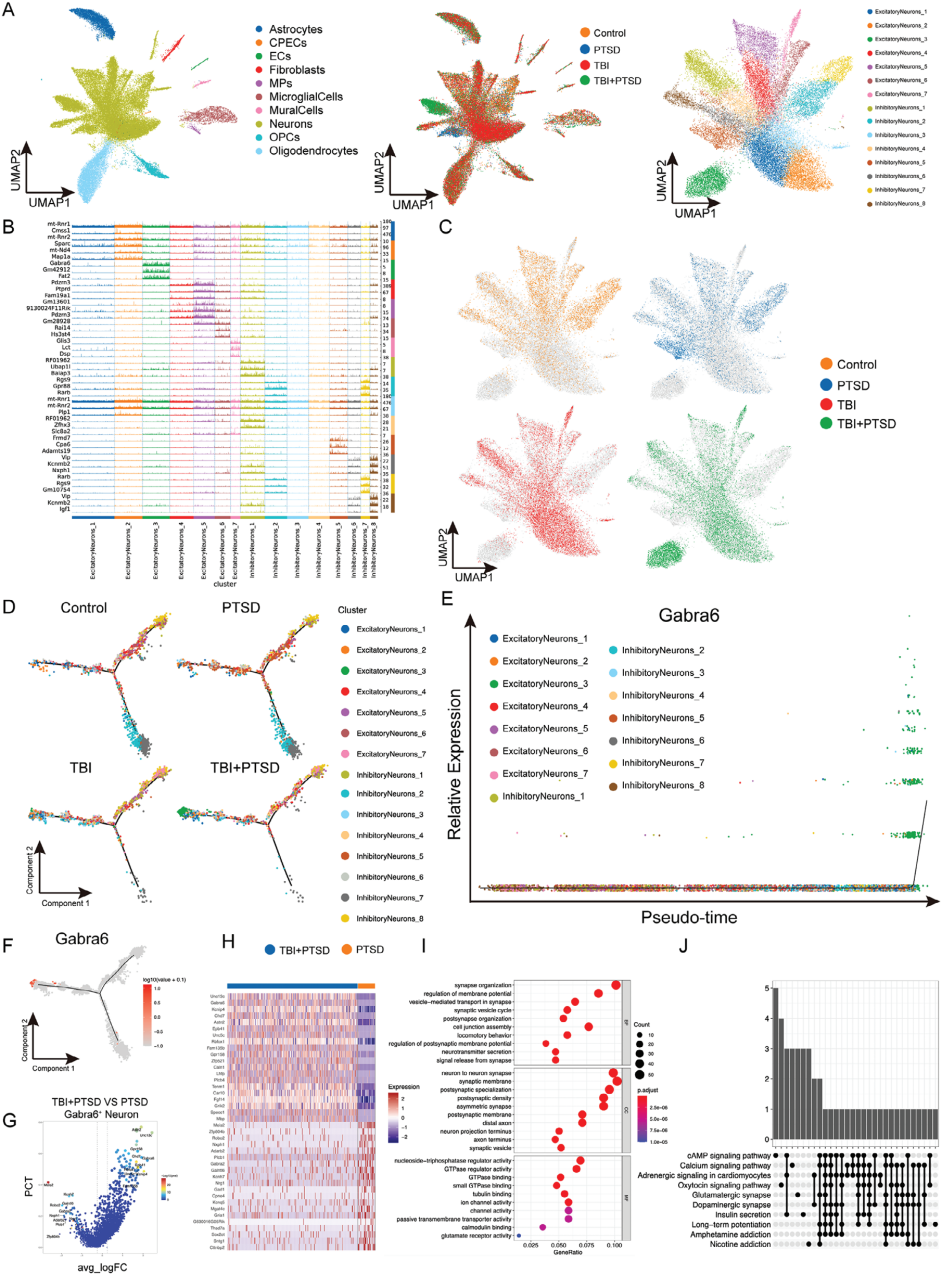
The proportion of cells in Gabra6^+^ neurons rises in TBI‐induced PTSD. A) UMAP plots for cell subset classification for each group; Three mice per group were tested after mixing samples. n = 3; one experiment performed. B) Top3 markers were expressed in various types of cells. C) UMAP maps of excitatory neurons_3 in each group. D) Pseudotime analysis of neuronal subpopulations in each group. E) Analysis of Gabra6^+^ neurons on a pseudo‐timeline. F) The expression of the Gabra6 gene in the DDTree plot. G) Volcano plot of genes differentially expressed in Gabra6^+^ neurons between PTSD group and TBI‐induced PTSD groups. H) The upregulated genes of Gabra6^+^ neurons in each group. I) GO analysis of the result in panel H. J) KEGG analysis of the result in panel H.

To minimize intra‐group errors caused by individual differences in mice, samples of three mice per group were mixed and tested, as reported in the literature.^[^ ^21^, ^22^, ^23^, ^24^ ^]^ snRNA‐seq was conducted on the cortical samples from various groups of mice, isolating and identifying a total of 47344 cells across 10 distinct cell types, including astrocytes, choroid plexus epithelial cells, endothelial cells, fibroblasts, mononuclear phagocytes, microglial cells, mural cells, neurons, oligodendrocyte precursor cells and oligodendrocytes (**Figure** **2**A; Figure S2A, Supporting Information). In addition, there was no significant difference in the proportion of various types of cells and cell‐cell interaction between groups (Figure S2B–D, Supporting Information). Further analysis of cell subsets according to the expression levels of different markers in neurons (Figure 2B) showed that cortical gamma‐aminobutyric acid type A receptor subunit alpha6 (Gabra6)^+^ neurons were significantly increased in mice of TBI+PTSD group compared with that of other groups, suggesting that TBI may play a role in promoting PTSD formation through Gabra6^+^ neurons (Figure 2C). Pseudotime analysis showed that the increased Gabra6^+^ neurons may have been transformed from other types of neurons rather than completely independent cell populations. This suggests that TBI‐induced overexpression of Gabra6 in neurons may have affected PTSD formation in mice (Figure 2D–F; Figure S3, Supporting Information). GO and KEGG analysis of Gabra6^+^ neurons in the prefrontal cortex of mice in the PTSD group and TBI+PTSD groups revealed that differential genes were mainly associated with the composition of synapses between neurons and cAMP signaling (Figure 2G–J; Figure S2E, Supporting Information). These data suggest that cortical Gabra6^+^ neurons may be involved in the formation of TBI‐induced PTSD in mice.

### 2.3 Neuronal Overexpression/Knockdown of Gabra6 Promotes/Inhibits PTSD Formation In Vitro and In Vivo

**Figure 3:**
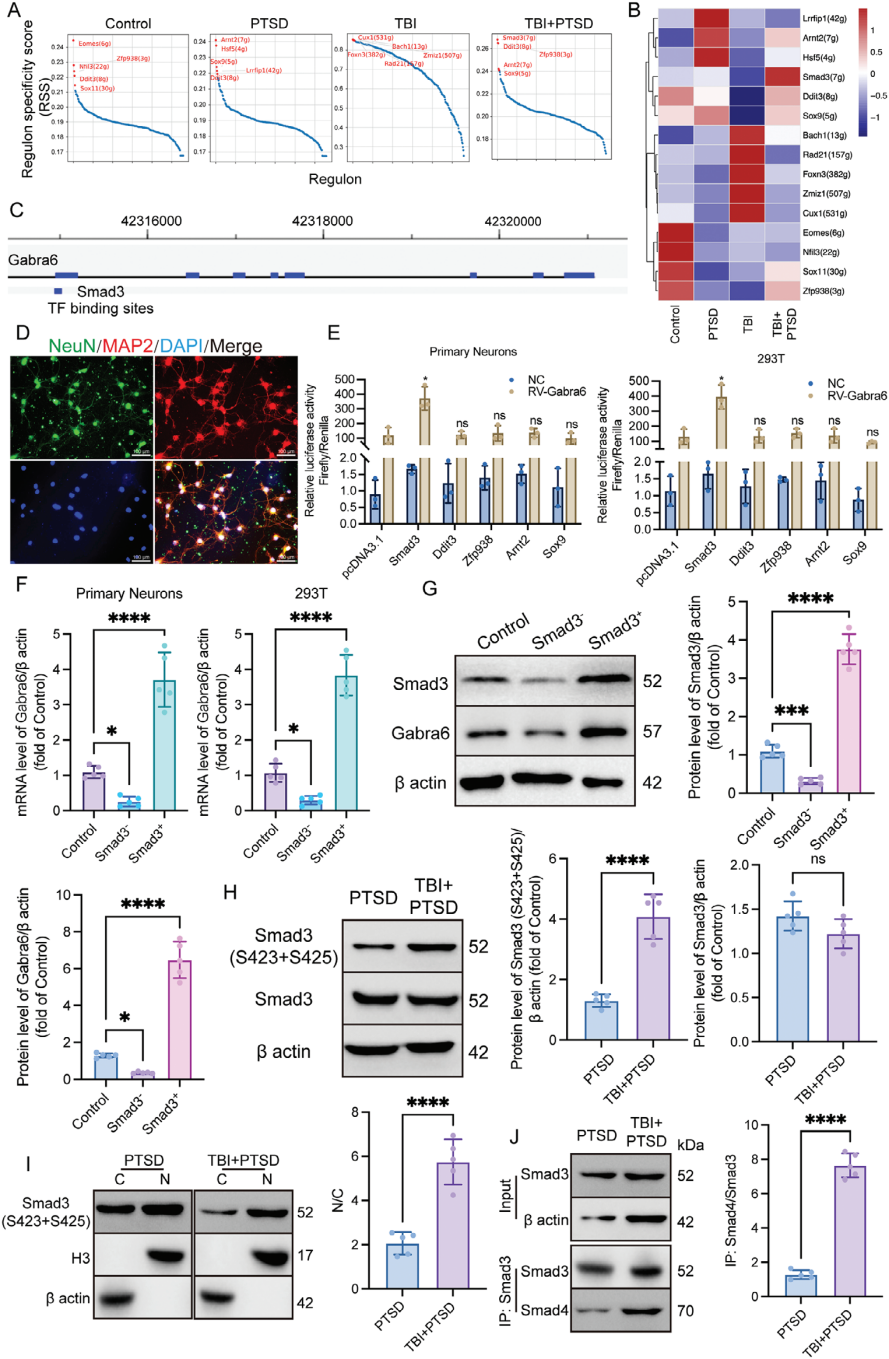
Overexpression of Gabra6 promotes PTSD formation in vitro and in vitro models. A) Representative trajectory plots for open field experiments; different modified mice after 2 W after PTSD modeling. B) Representative trajectory plots for elevated plus maze; different modified mice after 2 W after PTSD modeling. C) Quantification of Panel A and B. Open field experiments: Central/Total distance: F (2, 12) = 34.91, *P < 0.0001*, Central/Total time: F (2, 12) = 84.13, *P < 0.0001*; Elevated plus maze: Open arms/Total distance: F (2, 12) = 165.5, *P < 0.0001*, Open arms/Total time: F (2, 12) = 155.7, *P < 0.0001*. D) mRNA levels of IL‐1β, TNF‐α, SLC6A4, IL‐10, ERCC6L2, and GAD1 in different modified primary neurons. IL‐1β: F (6, 28) = 110.8, *P < 0.0001*; TNF‐α: F (6, 28) = 216.9, *P < 0.0001*; SLC6A4: F (6, 28) = 94.33, *P < 0.0001*; IL‐10: F (6, 28) = 53.93, *P < 0.0001*; ERCC6L2: F (6, 28) = 44.98, *P < 0.0001*; GAD1: F (6, 28) = 71.63, *P < 0.0001*. The data were analyzed using one‐way ANOVA (C and D), and all data are expressed as the mean ± standard deviation. ^*^p < *0.05*, ^**^p < *0.01*, ^***^p < *0.001*, and ^****^p < *0.0001* represent a statistically significant difference between the two groups. Each experiment was repeated three times.

To investigate whether Gabra6^+^ neurons could promote PTSD formation in mice, AAVs, and lentivirus were utilized to interfere with Gabra6 expression in mouse prefrontal cortical neurons and primary cortical neurons. The transfection efficiency and interference efficiency of AAVs and lentiviruses have been validated (Figure S4A,B, Supporting Information). The expression level of Gabra6 in the prefrontal cortex does not affect the behavioral phenotype of mice (Figure S4C, Supporting Information). After neurons stably expressed AAVs, mice, and cells were modeled for PTSD using the SPS method or Dex/Hcort treatment. The behavioral results showed that mice with neuronal overexpression of Gabra6 had more intense depression‐like and phobic behaviors, while Gabra6 knockdown mice had stronger motor and exploratory abilities after modeling than that of the PTSD group (**Figure** **3**A–C). Many studies have now reported that PTSD formation is associated with cerebral prefrontal cortex‐hippocampus‐amygdala circuits, so we examined the expression levels of PTSD‐related parameters in various regional tissues of mice. It was found that high Gabra6 expression resulted in up‐regulation of IL‐1β, TNF‐α, and SLC6A4 expression in both the prefrontal cortex and hippocampus, whereas Gabra6 knockdown promoted down‐regulation of IL‐10, ERCC6L2, and GAD1 expression (Figure S4D, Supporting Information). In addition, we also detected the above inflammation‐related and depression‐related indicators using lentiviral interference with Gabra6 expression in a primary neuronal model of PTSD induced by DEX or Hcort in vitro and obtained the same experimental results as in vivo (Figure 3D). These data illustrate that overexpression of Gabra6 in cortical neurons is a critical factor in promoting PTSD formation in mice.

### 2.4 Highly Expressed Gabra6 is Regulated by Transcription Factors of the Smad3/4 Complex

**Figure 4:**
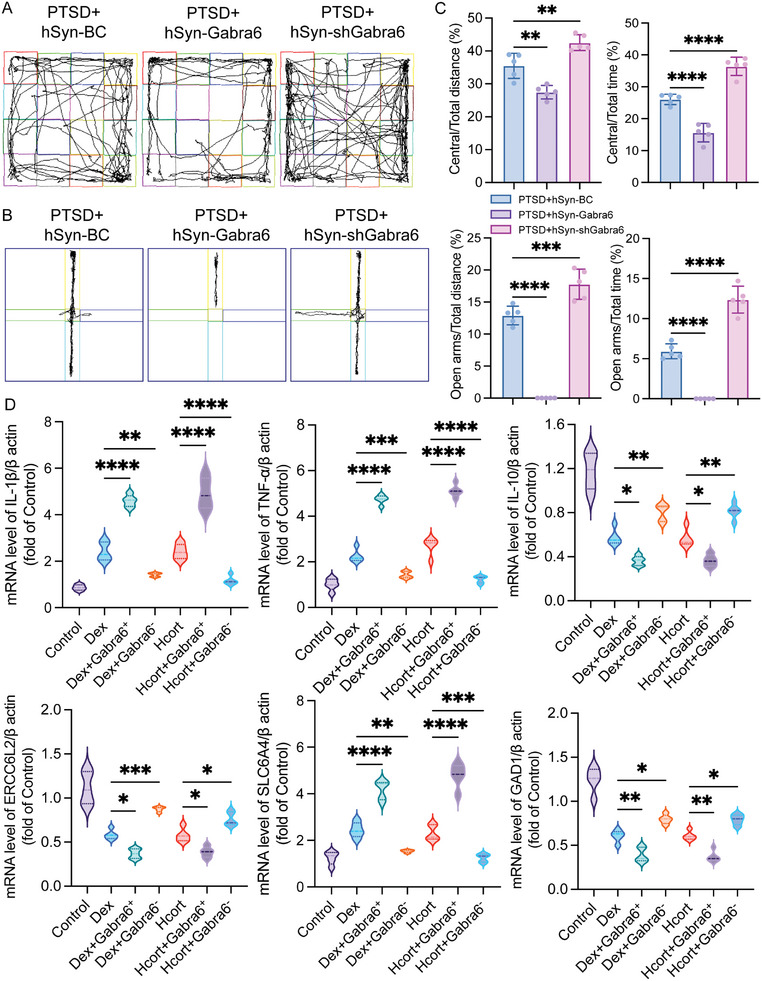
The expression of Gabra6 was regulated by the Smad3/4 complex. A) The regulon‐specific score scatter plot for Gabra6^+^ neurons in each group. B) The highest average expression of transcription factor in Gabra6^+^ neurons by pySCENIC analysis. C) GTRD was used to analyze potential binding sites and abilities of Gabra6 and Smad3. D) Representative immunofluorescence results for markers NeuN and MAP2 in primary neurons. E) Dual‐luciferase assays were performed to examine the transcriptional regulation of Gabra6 by different transcription factors. Relative fluorescence intensity: Firefly luciferase/Renilla luciferase. Primary neurons: F_Interaction_ (5, 24) = 12.83, *P < 0.0001*. 293T: F_Interaction_ (5, 24) = 15.42, *P < 0.0001*. F) qPCR was performed to examine the regulation of Gabra6 transcription by differentially expressed Smad3. Primary neurons: F (2, 12) = 75.27, *P < 0.0001*. 293T: F (2, 12) = 125.8, *P < 0.0001*. G) WB was performed to detect the regulatory effect of different Smad3 expressions on Gabra6 protein. Smad3: F (2, 12) = 256.9, *P < 0.0001*. Gabra6: F (2, 12) = 162.4, *P < 0.0001*. H) Expression levels of Smad3 and its phosphorylated protein in the prefrontal cortex of mice in each group. Smad3(S423+S425): t = 8.091, df = 8, *P < 0.0001*. Smad3: t = 1.901, df = 8, *P = 0.0938*. I) Expression levels of phosphorylated Smad3 in the nucleus and cytoplasm. C: cytoplasm; N: nucleus. N/C: t = 7.198, df = 8, *P < 0.0001*. J) IP was performed to detect the binding ability of Smad3 and Smad4. t = 19.23, df = 8, *P < 0.0001*. The data were analyzed using two‐way ANOVA (E), one‐way ANOVA (F and G), or Student's *t* test (H, I, and J), and all data are expressed as the mean ± standard deviation. ^*^p < *0.05*, ^***^p < *0.001*, and ^****^p < *0.0001* represents a statistically significant difference between the two groups. ns, not significant. Each experiment was repeated three times.

To investigate the mechanism of high Gabra6 expression in neurons, the levels of transcription factor activation of snRNA‐seq results in each group were analyzed. The results showed that the activation level of patent factors and the number of transcription factors were significantly different among groups (Figure S5A, Supporting Information). Activation of Smad3, Ddit3, Zfp938, Arnt2, and Sox9 transcription was predominantly predicted in mice of the TBI +PTSD group (**Figure** **4**A,B). The GTRD database predicts potential binding sites for transcription factors Smad3 and Gabra6 (Figure 4C). To further investigate which transcription factor regulates Gabra6 expression, normal mouse cortical primary neurons were extracted and presented positive expression of markers NeuN and MAP2 (Figure 4D). Dual‐luciferase assays showed that overexpression of Smad3 promoted the expression of reporter plasmid Gabra6 fluorescence in primary neurons and 293T cells (Figure 4E). In addition, the lentiviral intervention of Smad3 expression positively regulates transcriptional (Figure 4F) and translational (Figure 4G) levels of Gabra6 in primary neurons. Members of the SMAD family of signal transduction molecules are components of key intracellular pathways,^[^ ^25^ ^]^ and Smad3 forms a complex with Smad4 after phosphorylation at its carboxyl terminus and translocases to the nucleus to regulate gene expression.^[^ ^26^ ^]^ Compared with that of the PTSD group, the phosphorylation level of Smad3 in the cerebral prefrontal cortex of mice in the TBI+PTSD group was significantly increased (Figure 4H), and the expression of Smad3 (S423 + S425) was also significantly increased in the cytoplasm/nucleus (Figure 4I). The IP experiment results showed that Smad3 and Smad4 had binding ability and formed more complexes in the cerebral prefrontal cortex of mice in the TBI+PTSD group (Figure 4J). In addition, behavioral results showed that compared with that of the PTSD group, in situ, stereotactic injection of Smad3 protein (sf9, His‐GST) into the prefrontal cortex before PTSD modeling significantly aggravated PTSD‐like depression and fear symptoms in mice, and the difference was not statistically different from that in mice of TBI+PTSD group (Figure S5B,C, Supporting Information). Meanwhile, we also extracted the prefrontal cortex of mice two weeks post‐model induction and found that the exogenously injected Smad3 protein was still present under long exposure conditions in WB (Figure S5D, Supporting Information). It also demonstrated the ability to bind with endogenous Smad4, indicating biological activity (Figure S5E, Supporting Information). These data suggest that the regulation of Gabra6 expression by Smad3 may be responsible for the increase of Gabra6^+^ neurons induced by TBI in PTSD mice.

### 2.5 Gabra6 Binds to Postsynaptic Density and Regulates cAMP Signaling Pathways

**Figure 5:**
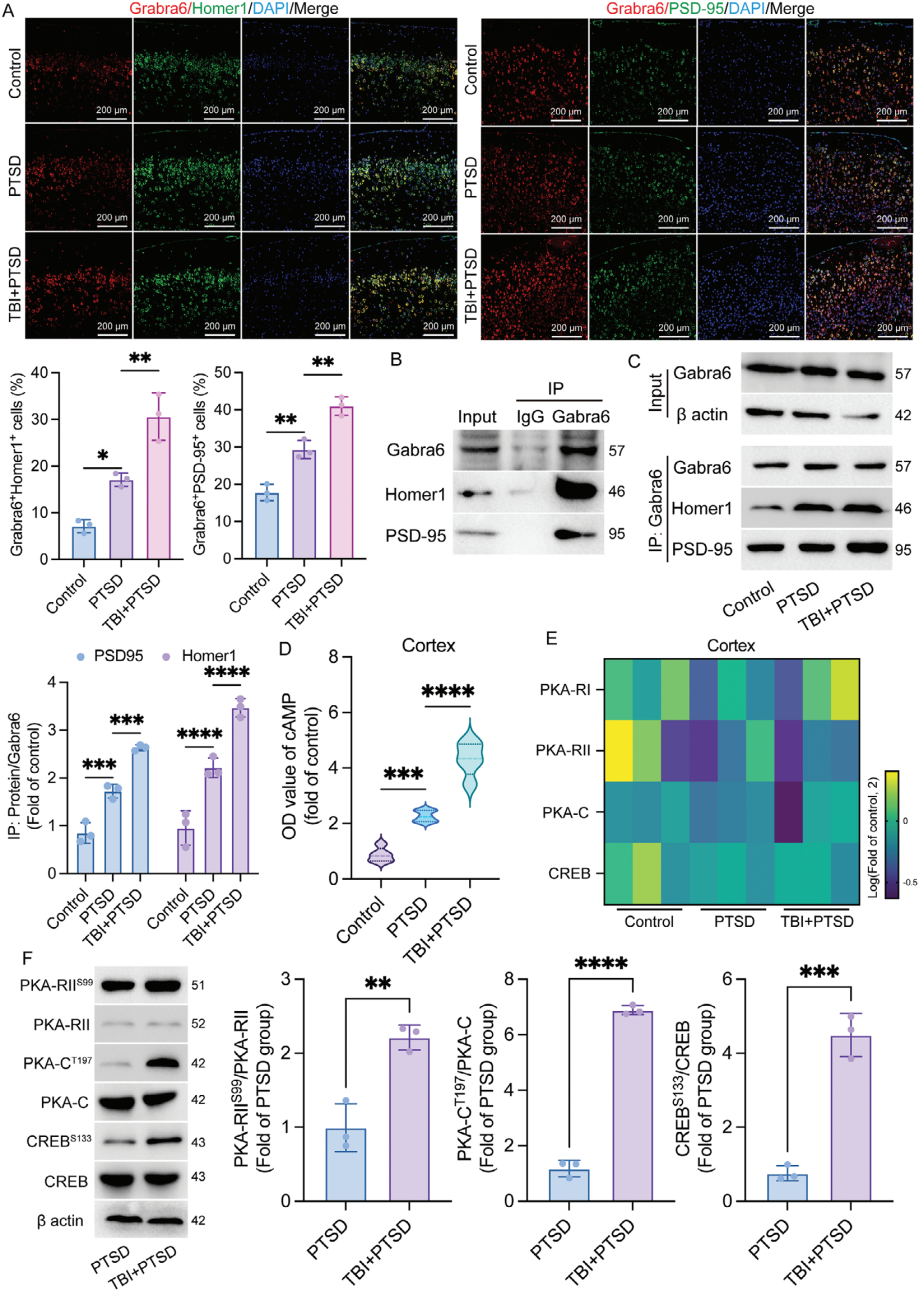
Gabra6 binds to Homer1 and PSD‐95 and regulates cAMP signaling pathways. A) IF was used to detect the co‐localization levels of Gabra6, Homer1, and PSD‐95 in the prefrontal cortex of mice in each group. Gabra6^+^Homer1^+^ cells: F (2, 6) = 42.22, *P = 0.0003*; Gabra6^+^PSD‐95^+^ cells: F (2, 6) = 71.31, *P <* *0.0001*. B) The IP assay was used to detect binding capacity between Gabra6, Homer1, and PSD‐95. C) Detection of differences in binding ability of proteins in the cerebral prefrontal cortex of each group. PSD‐95: F (2, 6) = 100.7, *P <* *0.0001*; Homer1: F (2, 6) = 68.56, *P <* *0.0001*. D) Elisa was used to detect the expression levels of cAMP in the prefrontal cortex of each group. F (2, 12) = 95.34, *P <* *0.0001*. E) Transcript levels of PKA‐RI, PKA‐RII, PKA‐C, and CREB in the cerebral prefrontal cortex of each group. F) Protein expression levels of key molecules of the cAMP signaling axis. PKA‐RII^S99^/PKA‐RII: t = 5.797, df = 4, *P = 0.0044*; PKA‐C^T197^/PKA‐C: t = 29.37, df = 4, *P <* *0.0001*; CREB^S133^/CREB: t = 10.46, df = 4, *P = 0.0005*. The data were analyzed using one‐way ANOVA (A, C, and D) or Student's *t*‐test (F), and all data are expressed as the mean ± standard deviation. ^*^p < *0.05*, ^**^p < *0.01*, ^***^p < *0.001*, and ^****^p < *0.0001* represent a statistically significant difference between the two groups. Each experiment was repeated three times.

The snRNA‐seq results suggest that differential gene expression in the prefrontal cortex of mice in the TBI +PTSD group and PTSD group is associated with synapse formation and function, as well as cAMP signaling pathways (Figure 2I,J). Homer1 and PSD‐95 are classical postsynaptic dense substances that mainly maintain the normal organization and function of synapses. The analysis of snRNA‐seq data indicates that there is no significant difference in the expression of nuclear transcripts of PSD‐95 and Homer1 between the groups (Figure S6A, Supporting Information). This finding aligns with the semi‐quantitative immunofluorescence results observed at the protein level (**Figure** **5**A). In addition, the results of the immunofluorescence co‐staining show that higher levels of Homer1 and Gabra6 co‐localization in the prefrontal cortex of mice in TBI+PTSD group compared to that of the PTSD group, although PSD‐95 co‐localized with Gabra6 as well (Figure 5A). The IP assay was used to further verify the presence of binding capacity, and it showed that Gabra6 is bound to both Homer1 and PSD‐95 (Figure 5B). Based on our prior experimental findings, Hcort was chosen for in vitro PTSD modeling, which was combined with a stretching system to establish an in vitro TBI+PTSD group. Further results showed that in both in vitro and in vivo models, the ability of Gabra6 to bind to both Homer1 and PSD‐95 was higher in the TBI+PTSD group when compared with that of the PTSD group, but the level of binding to Homer1 changed more significantly than that of PSD‐95 (Figure 5C; Figure S6B, Supporting Information). We also examined changes in expression levels of key molecules of the cAMP signaling axis. Elisa results showed that the expression level of cAMP in the TBI+PTSD group increased significantly in the cerebral prefrontal cortex both in vivo and in vitro. (Figure 5D; Figure S6C, Supporting Information) and did not change significantly in the hippocampal tissue (Figure S6D, Supporting Information) when compared with that of the PTSD group. The expression levels of PKA‐RI, PKA‐RII, PKA‐C, and CREB, which are key components downstream of the signaling axis, were further examined, and results of qPCR showed that the transcript levels of these molecules in the prefrontal cortex and hippocampus were not significantly different among groups (Figure 5E; Figure S6E, Supporting Information). However, phosphorylation levels of PKA‐RII, PKA‐C, and CREB were significantly different among groups in mouse prefrontal cortex and in vitro primary neuronal models, and the expression of PKA‐RII^S99^, PKA‐C^T197,^ and CREB^S133^ were significantly increased in TBI+PTSD models (Figure 5F; Figure S6F, Supporting Information). These results illustrate that Gabra6 may play a pro‐PTSD role by regulating the expression of key molecules of the downstream cAMP signaling axis after binding Homer1 in TBI‐induced PTSD models in vivo and in vitro. These results imply that Gabra6 binds to Homer1, but not PSD‐95, and plays an important role during cAMP signaling activation as well as TBI‐induced PTSD formation in primary neurons in vitro.

### 2.6 Knockdown of Homer1, but not PSD‐95, Suppressed cAMP Signaling and Improved PTSD Formation In Vitro

**Figure 6:**
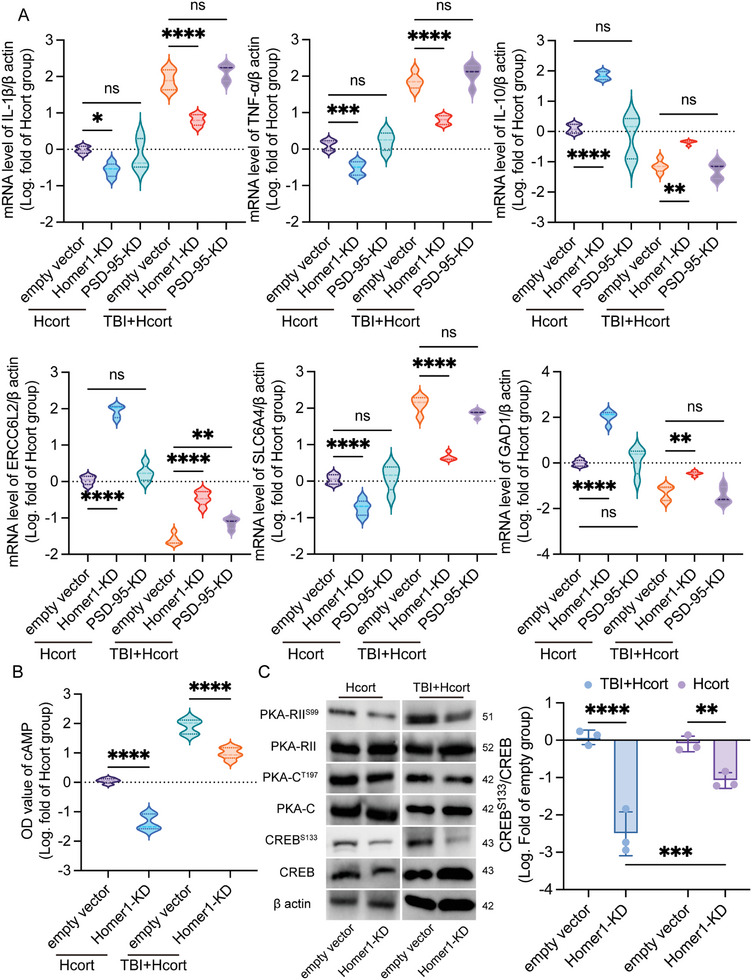
Knockdown of Homer1 suppressed cAMP signaling and improved PTSD formation in vitro. A) mRNA levels of IL‐1β, TNF‐α, SLC6A4, IL‐10, ERCC6L2, and GAD1 in different modified primary neurons. IL‐1β: F_Interaction_ (2, 24) = 8.145, *P = 0.0020*; TNF‐α: F_Interaction_ (2, 24) = 4.763, *P = 0.0181*; IL‐10: F_Interaction_ (2, 24) = 8.409, *P = 0.0017*; ERCC6L2: F_Interaction_ (2, 24) = 24.65, *P < 0.0001*; SLC6A4: F_Interaction_ (2, 24) = 6.835, *P = 0.0045*; GAD1: F_Interaction_ (2, 24) = 9.201, *P = 0.0011*. B) Elisa was used to detect the expression levels of cAMP in each group. F_Interaction_ (1, 16) = 6.930, *P = 0.0181*. C) Protein expression levels of key molecules of the cAMP signaling axis. CREB^S133^/CREB: F_Interaction_ (1, 8) = 16.39, *P = 0.0037*. The data were analyzed using two‐way ANOVA (A, B, and C), and all data are expressed as the mean ± standard deviation. ^*^p < *0.05*, ^**^p < *0.01*, ^***^p < *0.001*, and ^****^p < *0.0001* represent a statistically significant difference between the two groups. ns, not significant. Each experiment was repeated three times.

To investigate the effect of Homer1 and PSD‐95 on Gabra6 in PTSD formation, primary neuronal cell lines with knockdown of Homer1 and PSD‐95 were generated. qPCR results showed that knockdown of Homer1 improved the expression of inflammation‐related markers IL‐1β, TNF‐α, IL‐10, and depression‐related markers ERCCL2, SLC6A4, and GAD1 in primary neurons, both in the PTSD group and in the TBI+Hcort group. However, there was no significant statistical difference in these parameters after the knockdown of PSD‐95 compared with that of the blank virus group (**Figure** **6**A). We further examined the protein expression levels of cAMP signaling molecules in each group of cells, and the results of Elisa revealed that Homer1 knockdown could significantly reduce cAMP expression in each group, regardless of the presence of TBI factors (Figure 6B). Similarly, we observed that PKA‐RII^S99^, PKA‐C^T197^, and CREB^S133^ expression levels were down‐regulated in both groups after the knockdown of Homer1 at the protein level, but in the TBI +PTSD group, knockdown of Homer1 modulated protein decline more strongly than that of PTSD group (Figure 6C; Figure S7A, Supporting Information). These results reveal that Homer1 knockdown, but not PSD‐95, suppressed cAMP signaling and improved PTSD formation in vitro.

### 2.7 Conditional Knockout of Homer1 Suppresses PTSD Formation and cAMP Signaling Activation Following TBI

**Figure 7:**
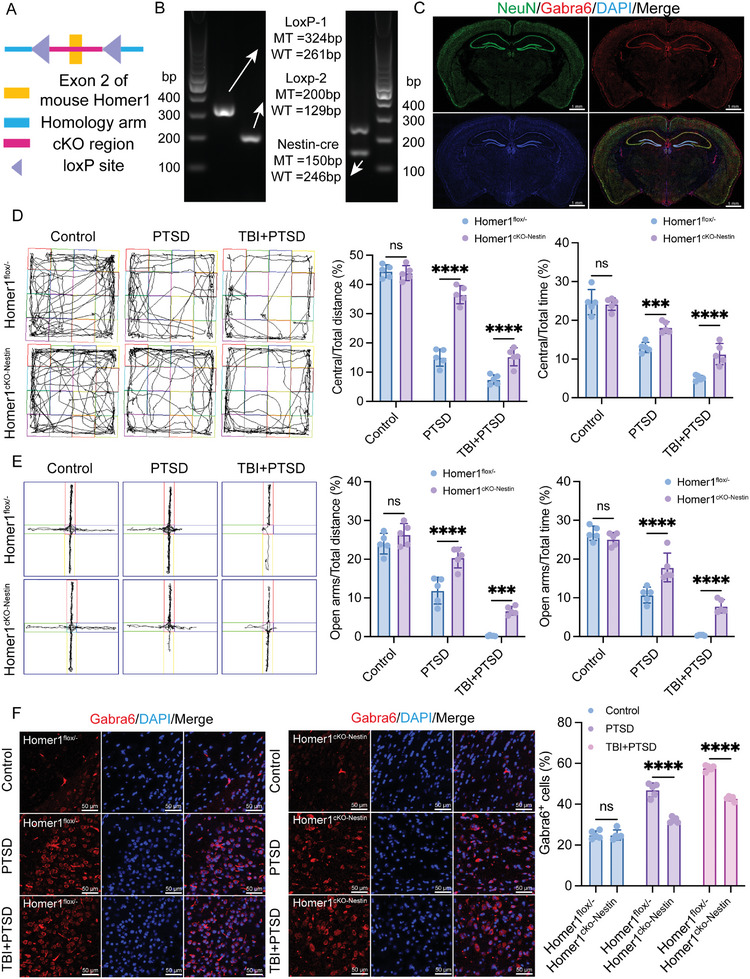
Conditional knockout of Homer1 suppresses PTSD formation. A) Schematic diagram of FLOX sites. B) Agarose gel electrophoresis was used to identify mouse genotypes. C) Gabra6 and NeuN co‐localization was detected by immunofluorescence. D) Representative trajectory plots for open field experiments; different modified mice after 2 W after PTSD modeling. Central/Total distance: F_Interaction_ (2, 24) = 44.96, *P < 0.0001*; Central/Total time: F_Interaction_ (2, 24) = 7.867, *P = 0.0024*. E) Representative trajectory plots for elevated plus maze; different modified mice after 2 W after PTSD modeling. Open arms/Total distance: F_Interaction_ (2, 24) = 4.179, *P = 0.0277*; Open arms/Total time: F_Interaction_ (2, 24) = 14.49, *P <* *0.0001*. F) IF was used to detect the expression of Gabra6 in the prefrontal cortex of mice in each group. F_Interaction_ (2, 24) = 41.60, *P <* *0.0001*. The data were analyzed using two‐way ANOVA (D, E, and F), and all data are expressed as the mean ± standard deviation. ^***^p < *0.001* and ^****^p < *0.0001* represent a statistically significant difference between the two groups. ns, not significant. Each experiment was repeated three times.

To further test the role of Homer1 in the development of PTSD following TBI in mice, neuronal conditional knockout Homer1 transgenic mice were generated. Exon 2 of mouse Homer1 was knocked out (**Figure** **7**A) and gene identification was performed using agarose gel electrophoresis (Figure 7B). Immunofluorescence results showed that Gabra6 was normally expressed in Homer1^cKO‐Nestin^ mice without regional specificity, suggesting that the knockdown of Homer1 did not affect Gabra6 expression (Figure 7C). Consistent with previous results, Homer1^cKO‐Nestin^ and Homer1^flox/−^ mice in the TBI+PTSD group had more pronounced behavioral performance than mice in the PTSD group 15 days after modeling. In addition, Homer1^cKO‐Nestin^ mice showed less depressive and fearful symptoms than controls, regardless of the model (Figure 7D,E). Extraction of mouse cortical tissue for qPCR revealed that intergroup differences in the expression of IL‐1β, TNF‐α, IL‐10, ERCCL2, SLC6A4, and GAD1 were more significant in Homer1^flox/−^ mice than in Homer1^cKO‐Nestin^ (Figure S8A, Supporting Information). This suggests that Homer1^cKO‐Nestin^ mice are more mildly characterized for PTSD after modeling. Previous results showed elevated expression of Gabra6 in the TBI+PTSD group (Figure 5A), and we observed the same trend of Gabra6 expression in the prefrontal cortex of Homer1^flox/−^ mice. Although Gabra6 expression levels were also observed to rise following PTSD in the prefrontal cortex of Homer1^cKO‐Nestin^ mice, the fold rise was lower than in Homer1^flox/−^ mice (Figure 7F). In addition, the results of Elisa and WB also showed that the intergroup differences in cAMP content (Figure S8B, Supporting Information) and expression levels of PKA‐RII^S99^, PKA‐C^T197^, and CREB^S133^ (Figure S8C, Supporting Information) in cortical brain tissue were more significant in Homer1^flox/−^ mice compared with Homer1^cKO‐Nestin^ mice. These data illustrate that conditional knockout of Homer1 suppresses PTSD formation and cAMP signaling activation following TBI.

### 2.8 The 218^th^ Glu in the Gabra6 Sequence May Play an Important Role in Inducing the Formation of PTSD

**Figure 8:**
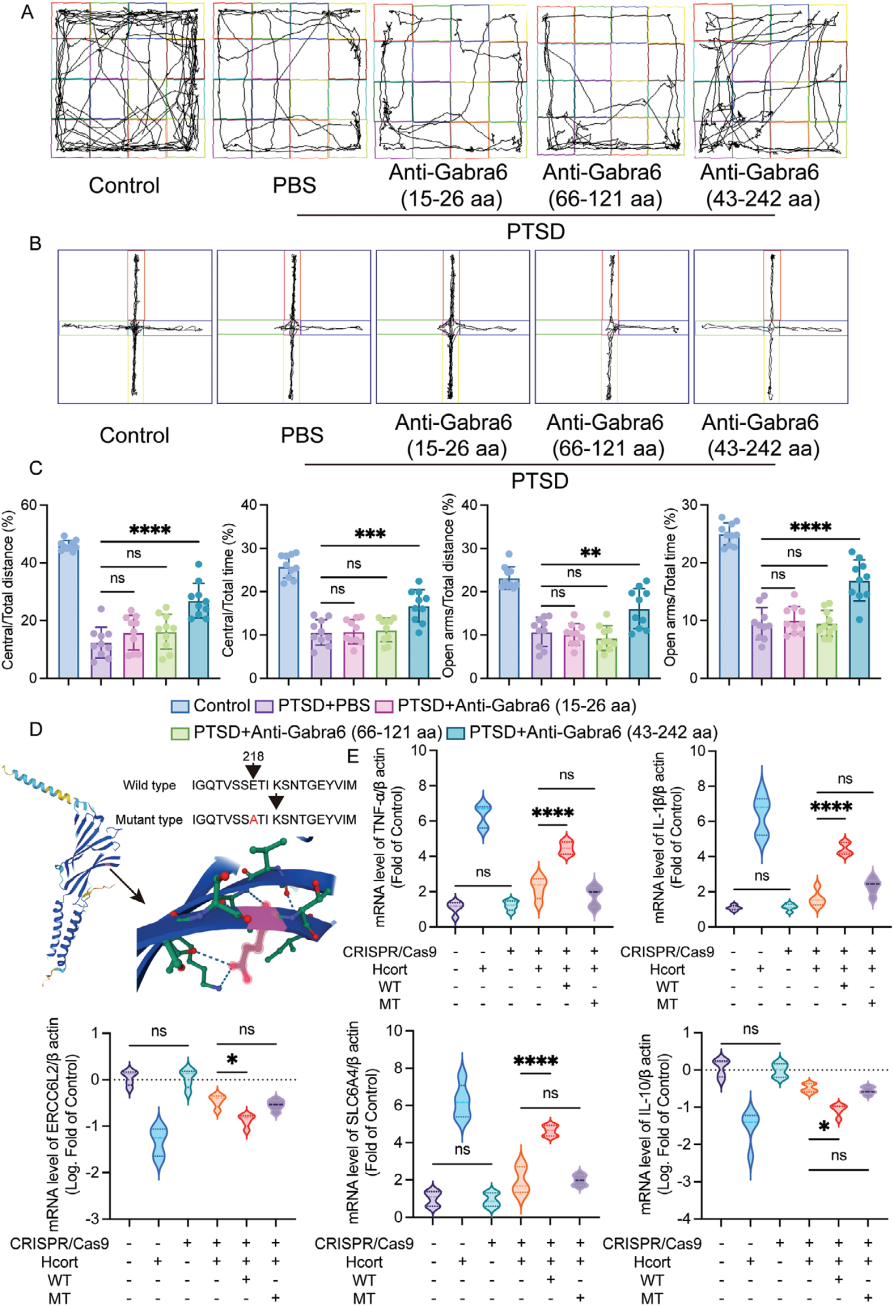
The 218^th^ Glu in the Gabra6 sequence may play an important role in inducing the formation of PTSD. A) Representative trajectory plots for open field experiments; different modified mice after 2 W after PTSD modeling. Antibody (1 µg) was dissolved in 2 µL PBS and injected orthotopically into the prefrontal cortex after PTSD modeling for three consecutive days. B) Representative trajectory plots for elevated plus maze; different modified mice after 2 W after PTSD modeling. C) Quantification of Panel A and B. Open field experiments: Central/Total distance: F (4, 45) = 67.40, *P <* *0.0001*; Central/Total time: F (4, 45) = 48.04, *P <* *0.0001*; Elevated plus maze: Open arms/Total distance: F (4, 45) = 31.76, *P <* *0.0001*; Open arms/Total time: F (4, 45) = 67.07, *P <* *0.0001*; D) 3D schematic of Gabra6 protein mutation sites. E: mRNA levels of IL‐1β, TNF‐α, SLC6A4, IL‐10, and ERCC6L2 in different modified primary neurons. TNF‐α: F (5, 24) = 92.01, *P <* *0.0001*; IL‐1β: F (5, 24) = 76.10, *P <* *0.0001*; ERCC6L2: F (5, 24) = 41.12, *P <* *0.0001*; SLC6A4: F (5, 24) = 75.66, *P <* *0.0001*; IL‐10: F (5, 24) = 32.00, *P <* *0.0001*; The data were analyzed using one‐way ANOVA (C and E), and all data are expressed as the mean ± standard deviation. ^*^p < *0.05*, ^**^p < *0.01*, ^***^p < *0.001*, and ^****^p < *0.0001* represent a statistically significant difference between the two groups. ns, not significant. Each experiment was repeated three times.

Polymorphisms encoded by GABRA6 are important risk factors for the development of idiopathic generalized epilepsy and a case of Dravet syndrome associated with GABRA6 (Glu218Ala) mutation has been reported. To investigate whether amino acid Glu at position 218 in the Gabra6 protein plays a key role in PTSD formation, mice were injected with Gabra6 monoclonal antibodies with different binding sites during the anesthetic phase of PTSD modeling. Mice injected with monoclonal antibodies at positions 15–26aa and 66–121aa showed no significant difference in behavioral outcomes compared with mice injected with PBS, but mice injected with antibodies at positions 43–242 significantly improved PTSD‐like depression and fear symptoms (**Figure** **8**A–C). In addition, in an in vitro model, 43–242aa monoclonal antibodies were added to the cell culture medium simultaneously with Hcort, and it was found that the expression of inflammation‐related markers IL‐1β, TNF‐α, IL‐10 and depression‐related markers ERCCL2, SLC6A4, and GAD1 were alleviated in the 43–242aa monoclonal antibodies treatment group compared with either the Hcort group or the TBI combined with Hcort treatment group (Figure S9A, Supporting Information). In addition, TERT lentivirus was used to first induce unlimited proliferation of primary neurons, and then Gabra6 knockout primary neurons were successfully constructed using CRISPR/Cas9 technology based on the 3D protein structure prediction of Gabra6 (Figure 8D). The results of qPCR showed that knockout of Gabra6 in normal cells did not affect the expression changes of IL‐1β, TNF‐α, IL‐10, ERCCL2, SLC6A4, and GAD1. Knockout of Gabra6 in the Hcort model group also did not affect the changes in the above PTSD‐related parameters, and further transfection of wild‐type but not mutant Gabra6 plasmid could induce cells again for PTSD modeling (Figure 8E; Figure S9B, Supporting Information). These data illustrate that the 218th Glu in the Gabra6 sequence may play an important role in inducing the formation of PTSD.

### 2.9 Mendelian Randomized Studies Revealed No Causal Relationship Between PTSD and Cortical Structure

To investigate the causal relationship between PTSD and cerebral cortical structures defined by surface area and thickness measured by magnetic resonance imaging using a Mendelian randomized study (Figure S10A, Supporting Information). A total of 17 index SNPs were chosen for genetic prediction of PTSD. The F statistics associated with these genetic instruments exceeded the conventional threshold of 10, signifying robust instrument strength.^[^ ^27^ ^]^ At the global level, PTSD had no causal relationship with the global SA and TH (β_SA_ = 67.43 mm^2^, SE_SA_ = 381.82, P_SA_ = 0.86; β_TH_ = 0.001 mm, SE_TH_ = 0.003, P_TH_ = 0.57). PTSD exhibited a trend toward reduced thickness of the Pericalcarine without global weighted (β = −0.007 mm, 95% CI: −0.01 to −0.0004 mm, P = 0.036), albeit failing to attain statistical significance following Bonferroni correction. However, this trend was not evident upon global weighting assessment (β = −0.004 mm, 95% CI: −0.01 to 0.001 mm, P = 0.13) (Figure S10B, Supporting Information).

## 3 Discussion

In the present study, we employed the BST‐I type biological shock tube to establish a shockwave‐induced TBI model and initially integrated it with the SPS method to create a comprehensive PTSD model. It is revealed that exposure to a 4.5 MPa intensity shock wave leads to significant advancement of PTSD. Analysis of cortical snRNA‐seq data unveiled a notable increase of Gabra6^+^ neurons during the progression of TBI‐induced PTSD. Both in vivo and in vitro experiments substantiated that heightened Gabra6 expression in neurons exacerbates the development of PTSD. Mechanistically, the upregulation of Gabra6 is tightly controlled by the Smad3/4 complex transcription factors. Furthermore, Gabra6 interacts with the postsynaptic density protein Homer1, thereby modulating the cAMP signaling pathway. Knocking out Homer1 conditionally impedes the onset of PTSD after TBI and the activation of the cAMP signaling cascade. Subsequent investigations highlighted the potentially pivotal role of the 218th Glu residue within the Gabra6 sequence in driving PTSD formation.

In practical scenarios, individuals afflicted by shockwave‐related injuries undergo injury incidents consciously, maintaining a normal perception of individuals’ external environment both before and after the injury. This conscious experience serves as a crucial external stressor contributing to the development of PTSD following shockwave‐induced TBI.^[^ ^28^, ^29^ ^]^ The pivotal aspect of PTSD formation hinges on the stress response to traumatic incidents, necessitating a specific process of trauma recall.^[^ ^30^, ^31^, ^32^ ^]^ However, ethical considerations in animal experimentation mandate that all animals undergo injury modeling under anesthesia, precluding them from fully experiencing the traumatic event and thereby hindering the formation of trauma memory and a stress‐related experimental foundation. Consequently, animals solely exposed to shockwave‐induced brain injuries face challenges in exhibiting typical PTSD symptoms. To address this limitation, our experimental design distinguishes shockwave‐induced brain injury and PTSD as two separate intervention factors. While shockwave‐induced brain injury entails the physical damage process affecting brain tissue and driving consequential alterations, PTSD modeling aims to replicate the traumatic stress experience, providing conscious animals with a simulated traumatic event encounter. Through the combined effects of these models, insights into whether shockwave‐induced TBI diminishes tolerance to PTSD formation and possibly triggers the onset of PTSD may be gleaned.

In addition to the above reasons, the PTSD mice induced by the composite model also have additional advantages. Although environmental events can clearly trigger PTSD, only a small number of trauma survivors actually develop the disorder. Researchers often use methods like trauma‐induced (TBI)^[^ ^33^ ^]^ or stress‐induced (SPS) models^[^ ^34^ ^]^ to create PTSD mice. While these factors can independently induce PTSD, single‐factor models struggle to accurately replicate the disease progression seen in PTSD patients in society. This has led to a search for risk factors that increase the likelihood of developing PTSD. Researchers have employed multi‐factor models to study PTSD^[^ ^35^, ^36^ ^]^ and have discovered gene‐environment (G×E) interactions underlying PTSD symptoms,^[^ ^37^ ^]^ underscoring the importance and necessity of multi‐factor PTSD models. In our experimental design, we utilized a combined TBI and SPS model to investigate the interaction between trauma and stress (TxS) that underlies PTSD symptoms. Our results demonstrate that while any single factor can induce PTSD‐like behaviors in mice, the symptoms are more severe when both factors are combined. Mechanistically, an increase in Gabra6^+^ neurons was observed only in the TBI+PTSD group, indicating a unique phenotype and mechanism for PTSD induced by TxS factors. Furthermore, in our comparative experimental design, we focused on the role of shockwave‐induced TBI in further promoting PTSD. Consequently, our subsequent analyses concentrated on the differences between the PTSD and TBI+PTSD groups to explore the additive effects and specific mechanisms of shockwave‐induced TBI trauma under normal stress conditions.

In our experimental findings, we observed notable similarities in the behavioral responses exhibited by mice in the TBI group (subjected to 4.5 MPa intensity) and PTSD group. Mice from the 4.5 Mpa TBI cohort displayed favorable outcomes in both the open field and elevated plus maze tests at the two‐week mark following shockwave modeling. Moreover, all subjects demonstrated a recovery in behavioral patterns approximately one‐month post‐modeling, suggesting that a singular 4.5 MPa TBI event can trigger PTSD symptoms, which aligns with the conventional behavioral presentations of PTSD reported in existing literature. Conversely, concerning the pathological analyses, mice in the TBI group showed signs of cortical neuronal damage, apoptosis, and activation of microglial cells, with these abnormalities demonstrating a positive correlation with the severity of the TBI. Notably, mice exposed to TBI induced by 5.5 MPa shockwaves exhibited more severe gross and pathological injuries a month later. This implies that the behavioral manifestations arising from high‐intensity shockwave injuries in mice may not be exclusively linked to a PTSD diagnosis but likely involve disturbances in the intrinsic behavioral regulatory mechanisms of the subjects. Moreover, the establishment of a composite TBI + PTSD model revealed that the behavioral responses of mice in the TBI + PTSD group surpassed those in either the PTSD or TBI group. This suggests that both the initial traumatic event and subsequent stressors operate as independent factors in the development of PTSD, and further substantiates the fidelity of the constructed TBI + PTSD composite model. Drawing from these insights, it is recommended that where there exists a possibility of PTSD occurrences post‐injury in patients or military personnel, prompt relocation to secure environments to mitigate exposure to mixed or hostile surroundings may effectively reduce the likelihood of PTSD incidence.

Presently, the cell model for PTSD remains underdeveloped, with research indicating the utilization of induced pluripotent stem cell models to explore PTSD mechanisms. To mimic stress responses that trigger PTSD, scientists expose induced pluripotent stem cell‐derived neurons to the stress hormone hydrocortisone, a synthetic variant of the body's cortisol involved in the “fight or flight” response.^[^ ^37^ ^]^ Gene characteristics identified in experimental cells are notably present in brain samples from post‐trauma PTSD patients, signifying that stem cell models offer a relatively accurate representation of brain events in living patients, facilitating accelerated PTSD diagnosis and treatment.^[^ ^37^, ^38^ ^]^ Our study findings imply that the target is not associated with pluripotent stem cells, prompting us to explore the feasibility of using primary neurons as a substitute for pluripotent stem cells in in vitro PTSD modeling. While our experiments employed primary neurons instead of induced pluripotent stem cells for the hormone stimulation model, we fortuitously observed significant gene expression changes associated with depression and emotional disorders after modeling, including ERCC6L2, SLC6A4, and GAD‐1, recognized as specific biological markers for anxiety or emotional disorders.^[^ ^39^, ^40^, ^41^ ^]^ Moreover, given the involvement of minor inflammatory responses in PTSD pathogenic mechanisms, we also investigated the expression of inflammatory factors IL‐1β, TNF‐α, and IL‐10,^[^ ^42^, ^43^ ^]^ yielding positive outcomes. Despite not every marker showing positive variations in each experiment, a holistic assessment of multiple indicators generally aligns with PTSD diagnostic criteria. The utilization of a robust stem cell or primary neuronal cell model presents an optimal avenue for in vitro drug screening across diverse patient cohorts. Furthermore, disparities in stress responses between PTSD and non‐PTSD cells may assist in predicting individuals at heightened risk of PTSD development.

The cortical‐hippocampal‐amygdala circuit plays a pivotal role in PTSD progression, with the amygdala as the brain's fear center and the hippocampus as its memory hub, and both are crucial in PTSD formation.^[^ ^44^, ^45^ ^]^ In comparison to the hippocampus and amygdala, the prefrontal cortex holds greater intricacy in PTSD genesis.^[^ ^46^ ^]^ Research by the NIH Holmes group unveiled that cortical‐amygdala presynaptic endocannabinoids targeting the CB1R receptor induce long‐term inhibition, reducing synaptic strength and facilitating fear extinction.^[^ ^47^, ^48^ ^]^ Additionally, the TRN‐Pom‐FrA thalamic‐cortical loop regulates excessive defensive behaviors in PTSD.^[^ ^49^ ^]^ The researchers employed lipid nanoparticles to target the catalytic subunit of protein phosphatase 6 for siRNA therapy, aiming to correct abnormalities in the thalamic circuit, rectify dysfunction in the thalamic‐cortical circuit, and address behavioral abnormalities associated with fear memory extinction.^[^ ^50^ ^]^ In our experiments, the prefrontal cortex, as the region most immediately impacted after shockwave exposure, formed the primary focus of our investigation. However, positive experimental outcomes were not attained in the hippocampus. These results suggest that the mechanism by which TBI exacerbates PTSD may differ from conventional neural circuits, such as prefrontal cortex‐hippocampus‐amygdala circuits. Furthermore, it is indispensable to employ neurophysiological techniques to explore downstream brain regions and signal‐receiving cells associated with cortical Gabra6^+^ neurons to unveil novel PTSD pathogenic mechanisms.

The gamma‐aminobutyric acid (GABA) type A receptor, an ionotropic receptor serves as a major neurotransmitter in the central nervous system. GABA activates the GABAA type A receptor selectively for Cl‐ channel opening, inducing neuronal hyperpolarization that inhibits neural signal transmission.^[^ ^51^ ^]^ Gabra6, a member of the GABAA type A receptor subunit family encoded by the gene located on chromosome 5q34, encodes the α6 subunit of the GABAA receptor. Current research mainly focuses on Gabra6 polymorphisms as significant risk factors for idiopathic generalized epilepsy,^[^ ^52^ ^]^ with limited exploration of Gabra6 in PTSD progression. The T1521C polymorphism in the GABRA6 gene is associated with specific personality characteristics as well as a marked attenuation in hormonal and blood pressure responses to psychological stress.^[^ ^53^ ^]^ A study by Luo et al. reported a case of GABRA6 (Glu218Ala) mutation‐associated Dravet syndrome. However, due to limited cases, no recommended treatments exist for this genetic variant.^[^ ^54^ ^]^ Our research is the first to demonstrate that Gabra6 overexpression in neurons promotes PTSD progression, with further investigations indicating the crucial role of the 218th amino acid in its pro‐PTSD effects. This discovery offers optimism for utilizing Gabra6‐based therapies in TBI‐induced PTSD. Furthermore, while we elucidated the reasons for heightened Gabra6 expression and its impact on PTSD at the protein level, Gabra6 fundamentally belongs to ion channels, necessitating further exploration to determine if its neuroelectrophysiological characteristics alter during the PTSD formation process.

To summarize, our investigation represents the first to elucidate the promotion effect of neuronal Gabra6 overexpression in the mouse prefrontal cortex on the progression of TBI‐induced PTSD. Introducing monoclonal antibodies targeting the 218th amino acid residue of Gabra6 could offer a promising avenue for the prevention and treatment of PTSD induced by similar traumas. These novel findings provide valuable mechanistic insights and support for the therapeutic and preventive strategies for TBI‐induced PTSD.

## 4 Experimental Section

### Animal and Experimental Grouping

All animal experiments were performed in accordance with protocols approved by the Institutional Ethics Committee of Xijing Hospital. All experimental procedures were approved by the Institutional Animal Care and Use Committee of Air Force Military Medical University. All animals were purchased from the Shanghai Model Organisms Center, Inc. (Shanghai, China). All mice were maintained in the same environment.

In animal experiments, mice were divided into four groups according to different treatments as follows: normally housed mice were set as the control group, mice with SPS alone were set as the PTSD group, mice with TBI alone were set as the TBI group, and mice with combined TBI and SPS group set as the TBI + PTSD group.

In cell experiments, normal cultured primary neurons were assigned to the control group, Hcort or Dex‐treated cells were assigned to the Hcort or Dex groups, cells subjected to traction modeling were assigned to the TBI group, and cells supplemented with Hcort or Dex hormones after traction modeling were assigned to the TBI + Hcort or TBI + Dex groups.

### Establishment of the TBI Model

C57BL/6J mice were used to establish the TBI model which was produced by the BST‐I type biological shock tube developed by the Army's characteristic medical center, with a 4 × 4 mm clamping film of aluminum diaphragm. The shock tube has a total length of 39.0 m and is composed of a driving section, an expansion section, a transition section, a testing section, an attenuation band and auxiliary equipment, an air compressor, a high‐pressure gas tank, etc. The shock tube adopts a double capsule structure. The driving section is 1.41 m long with an inner diameter of 0.348 m, the expansion section is 1.0 m long with an inner diameter of 0.348‐1 m. The transition section and test section are 24.0 m long, the attenuation band is 11 m long, and the inner diameter is 1 m. For brain TBI, the mice were placed in a restraint cage on a fixed frame to ensure that the mice maintained consistent orientation within the cage. The fixed frame was placed in the BST Type I biological shock tube at 28 m from the driving section, 10 cm in front of the end baffle of the test section, and the end was closed. When the animal was injured, the head and nose were facing toward the shock tube driving section, and the pressure in the driving section was 3.5, 4.5, and 5.5 MPa. These mice were designated as the TBI group.

### Stereotactic Injection

The prefrontal cortex of mice contains multiple nuclei. Based on literature reports,^[^ ^55^ ^]^ the most common and classical coordinates for viral and drug injections were selected. The coordinates, with bregma as the origin, are anterior‐posterior: +1.7 mm; mediolateral: 0.35 mm; and dorsal‐ventral: −2.5 mm. According to the experimental design, adeno‐associated virus (AAVs) was injected at this coordinate 3 weeks prior to modeling, with an injection speed of 2 µL/5 min. The injection concentration conditions for Smad3 protein and monoclonal antibodies were as follows: Protein/Antibody (1 µg) was dissolved in 2 µL of PBS and injected orthotopically into the prefrontal cortex after PTSD modeling for three consecutive days. The injection sequence was left brain first, followed by the right brain.

### In Vitro TBI Modeling

The cell culture was conducted in culture dishes specifically designed for a uniaxial cell stretching system (CELL&FORCE, China, Hangzhou), with cells allowed to fully adhere before the introduction of 10 µm of LPS into the medium to induce mild inflammation. The system was then set to apply sinusoidal wave stretching. One hour after stretching, the culture dishes were transferred to a standard incubation chamber for further cultivation. After 24 h, the cells were collected for subsequent experiments.

### The SPS Method is Used for PTSD Modeling

SPS was used as a mouse model of PTSD, and a series of behavioral assays were applied to assess the impact of SPS on reward.^[^ ^10^ ^]^ The mice were acclimatized to the animal room for ≈7 days. Initially, a hard plastic box was used to restrain the mice (with the limbs and trunk confined in the box, leaving the tail exposed) for a 2‐h period of restraint, followed by a forced swim at water temperatures of 20 to 24 °C for 20 min. Afterward, they were allowed a 15‐min rest period and then anesthetized with ether until they showed no resistance. The temperature and humidity during the restraint period were consistent with the normal breeding environment. After the stress procedure was completed, the mice were separated and returned to the animal room for breeding, designated as the PTSD group. For the TBI + PTSD group, mice were normally raised for one week after TBI before undergoing the SPS method for PTSD modeling.

### In Vitro PTSD Modeling

Although the disease‐relevant cell type (s) for PTSD remain unidentified, it was known that trauma‐induced perturbations in glucocorticoid signaling alter glutamatergic neural activity.^[^ ^56^, ^57^ ^]^ The literature reports that NGN2‐induced glutamatergic neurons were treated with dexamethasone (DEX) and hydrocortisone (Hcort) hormones in vitro to simulate PTSD conditions.^[^ ^37^ ^]^ Although the DEX‐induced genes did not exhibit any differential expression between donors with and without PTSD,^[^ ^37^, ^38^ ^]^ to treat primary progenitor cells with DEX (50 nm for 24 h) and HCort (1000 nm for 24 h) separately to explore the cellular model of PTSD was planed. Finally, the expression levels were assessed of inflammation‐related molecules IL‐1β, TNF‐α, IL‐10, and psychiatric disorder‐related markers ERCC6L2, SLC6A4, and GAD1 in cells to comprehensively evaluate the progression of the disease in the study of PTSD.

### The Construction and Identification of Transgenic Mice

The transgenic mice (Homer1^flox/flox^Nestin^Cre^) were purchased from Cyagen Biosciences Inc. One Step Mouse Genotyping Kit (Vazyme, China) was used for PCR experiments to identify the genotypes of the mice, with the procedures carried out according to the instructions provided by the reagent manufacturer. Identification of Homer1‐LoxP‐1 (mutant: 324 bp, wild type: 261 bp): F1: 5′‐AGC TGC TTG TAT AGT CTG AGT GC‐3′, R1: 5′‐GGC TTT CCT CAA TAA TGA ACT G‐3′; Homer1‐LoxP‐2 (mutant: 200 bp, wild type: 129 bp): F2: 5′‐GTT GCA GCA CTG TAT GAC TTG ATA A‐3′, R2: 5′‐CTT CAA TTC TAC TGC ATG GAC TGT‐3′. Identification of Nestin‐Cre (wild type: 246 bp; mutant: 150 bp): Primer 1 (wild type): 5‐TTG CTA AAG CGC TAC ATA GGA‐3′, Primer 2 (mutant): 5‐CCT TCC TGA AGC AGT AGA GCA‐3′, Primer 3 (common): 5‐GCCTTA TTG TGG AAG GAC TG‐3′.

### Open Field Test

The open field test (n = 20/group) was performed in a quiet environment and each experiment was completed in the same period. The open field box of the mice was 30 cm high, with a 75 cm bottom edge length and a white bottom surface. Open‐field experiments were performed sequentially according to the group and number of mice. The animals were placed inside the box at the center of the bottom surface and photographed and timed simultaneously. Shooting was stopped after 5 min of observation, and the inner wall and bottom of the square box were cleaned (75% alcohol solution) to avoid information left by the last animal (such as urine and olfactory cues) from affecting the subsequent test results. OpenField 2.8.5 software (Mobiledatum Co., Ltd, Shanghai, China) was used to calculate the total movement distance and the movement distance within the central area.

### Elevated Plus Maze

The experiments were performed in a quiet environment. Each experiment was run during the same time of day. The elevated plus maze test was performed sequentially according to the mouse group and number. The arm width, arm length, and closed arm height were 5, 35, and 15 cm, respectively and the maze was 50 cm above the ground. At the beginning of the experiment, mice were placed into the maze facing the closed arm from the central grid, and the activity was recorded over a 5 min period. After completion of the experiment, the mice were removed, the arms were cleaned, the olfactory cue was removed by spraying alcohol, and finally data analysis was performed using Highplus 2.8.1 software (Mobiledatum Co., Ltd, Shanghai, China).

### Primary Neuron Isolation and Culture

Neonatal C57BL/6J mice were used to extract primary neurons using a stereomicroscope. The culture dish was coated with 0.2 mg mL^−1^ of poly‐L‐lysine (Sigma–Aldrich) overnight at 37 °C, washed three times with sterile water, and placed in an incubator for use. The brain tissue was minced with sterile ophthalmic scissors and digested with 0.25% trypsin for 5 min at 37 °C before the brain tissue was centrifuged at 1000 rpm for 5 min.

For the extraction of primary neurons, the complete Dulbecco's Modified Eagle's Medium (DMEM, Gibco) was used for appropriate dilution and the cell suspension was made into a seed plate. After 4–6 h, DMEM was replaced with Neurobasal medium (Gibco) for primary neurons which contained 0.25% glutamine (Sigma‐Aldrich), 1% penicillin/streptomycin (Gibco), and 2% B‐27 supplement (Gibco). Neurons monolayers were obtained at the bottom of the dish after 7 days. The neurons were identified by morphological analysis and neuron‐specific enolase (NSE) staining.

### Gene Editing to Manipulate Protein Expression

Smad3 knockdown/overexpression lentivirus, Homer1 knockdown lentivirus, PSD‐95 knockdown lentivirus, Gabra6 knockdown/overexpression lentivirus, hSyn‐Gabra6 overexpression and knockdown adeno‐associated virus, Crisp/Casp9 single‐system knockout of Gabra6 lentivirus, Gabra6 wild‐type plasmid, Gabra6 mutant (E218A) plasmid were designed and constructed by Genechem Co., Ltd. and Hanbio Biotech Co., Ltd. According to the experimental design, different modified cells were transfected with plasmids using jet‐PRIME in vitro DNA transfection reagent (Polyplus).

### Double Luciferase Reporter Assay

The Gabra6 promotor firefly luciferase, transcription factor expression plasmid (Smad3, Ddit3, Zfp938, Arnt2, and Sox9), and Renilla reporter constructs were designed and constructed by Genechem (Shanghai, China). Plasmids were co‐transfected into primary neurons and 293T cells using the Polyplus/jetPRIME kit. Twenty‐four hours after transfection, the regulatory effects of the transcription factors and target gene promoters were detected using a dual‐luciferase assay kit (Promega, cat. no. E1910), and the experimental procedures were performed according to the manufacturer's instructions. Cells were analyzed in three wells per experiment to obtain average counts and in three independent biological replicates. The promoterless firefly‐luciferase vector pGL4.15 served as the negative control (NC). Renilla activity was used to normalize luciferase reporter activity.

### cAMP Content Detection

The cAMP Assay Kit (ab179459) was purchased from Abcam. The kit was utilized to detect the levels of cAMP in the samples. The experimental procedures were carried out strictly according to the manufacturer's instructions provided with the kit.

### Data Processing

For molecular biology experiments, three technical replicate experiments were performed for each mouse, and the data were averaged. The three mean values (n = 3/group) obtained from three mice in each group were used for statistical comparisons between groups. In vitro experiments, assays were performed on cells in three wells for each experiment to obtain an average count, and in three independent biological replicates.

For pathological experiments, one section from each of the three mice in each group was observed under immunofluorescence laser confocal microscopy, and three fields randomly selected from each section were used to quantify the detection indicators, and the data were averaged. The three mean values (n = 3/group) obtained for each group were used for statistical comparisons between groups.

Western blot (WB), Immunofluorescence (IF), Co‐immunoprecipitation (Co‐IP), Quantitative polymerase chain reaction (qPCR), Nuclear protein isolation, TUNEL assay, Flow cytometry, Cell viability assay, Nissl staining: All these experiments were performed as previously described.^[^ ^58^, ^59^, ^60^, ^61^, ^62^, ^63^ ^]^ Detailed information about the antibodies and kits used in this study is available in Table S2 (Supporting Information). Primers for qPCR were designed and synthesized by Beijing Tsingke Biotech Co., Ltd. The primer sequences used are listed in Table S3 (Supporting Information).

### Clinical Bioinformatics Analysis + R Language

The experiments above are described in detail in Supporting Information material and methods.

### Single Nucleus RNA‐seq (snRNA‐seq)

The experiments above are described in detail in Supporting Information material and methods.

### Statistical Analysis

Prism 8 for macOS software was used for the statistical analyses. Power analysis was used to ensure proper sample size. All values for each group were presented as mean ± SD. Parametric and nonparametric tests were used according to the homogeneity of variance. According to different comparison situations, statistical differences were analyzed using Student's t‐test or one‐way ANOVA, as appropriate, with Sidak's or Turkey's multiple comparisons test. *P* < 0.05 indicated that the difference was statistically significant.

## Conflict of Interest

The authors declare no conflict of interest.

## Supporting information



Supporting Information

## Data Availability

The data that support the findings of this study are available from the corresponding author upon reasonable request.
