# Supplementary material for: Single‐Nucleus RNA Sequencing Reveals That Gabra6+ Neurons in Prefrontal Cortex Promote the Progression of PTSD After Shockwave‐Induced TBI
Source: Adv Sci (Weinh). 2024 Dec 12;12(5):2407000. doi: 10.1002/advs.202407000 (PMC11792052; doi:10.1002/advs.202407000)
Supplement: Supplementary file 1 — Supporting Information [file ADVS-12-2407000-s001.docx]

Supporting Information

**Single-Nucleus RNA Sequencing Reveals that Gabra6^+^ Neurons in Prefrontal Cortex Promote the Progression of PTSD after Shockwave-Induced TBI**

Xiaowei Fei, Zehan Zhang, Ya-nan Dou, Weihao Lv, Hongqing Chen, Li Wang, Xin He, Wangshu Chao, Peng Luo* and Zhou Fei*

**Experimental Section**

*Single nucleus RNA-seq (snRNA-seq)*

*Single Nuclei isolation*: Cortical samples of three mice in each group (Mixed into an EP tube) were harvested and washed in pre-cooled PBSE (PBS buffer containing 2 mM EGTA). Nuclei isolation was carried out using GEXSCOPE® Nucleus Separation Solution (Singleron Biotechnologies, Nanjing, China) referring to the manufacturer's product manual. Isolated nuclei were resuspended in PBSE to 10^6^ nuclei per 400 μL, filtered through a 40μm cell strainer, and counted with Trypan blue. Nuclei enriched in PBSE were stained with DAPI (1:1,000) (TermoFisher Scientific, D1306). Nuclei were defined as DAPI-positive singlets.

*snRNA-seq library preparation*: The concentration of single nucleus suspension was adjusted to 3-4 × 10^5^ nuclei/mL in PBS. Single nucleus suspension was then loaded onto a microfluidic chip (GEXSCOPE® Single Nucleus RNA-seq Kit, Singleron Biotechnologies) and snRNA-seq libraries were constructed according to the manufacturer’s instructions (Singleron Biotechnologies). The resulting snRNA-seq libraries were sequenced on an Illumina instrument with 150 bp paired end reads.

*Single-cell RNA data processing and Analysis*: Seurat v 3.1.2 was used for quality control, dimensionality reduction and clustering. QC conditions were set as follows.:1) cells with gene count less than 200 or with top 2% gene count were excluded; 2) cells with top 2% UMI count were excluded; 3) cells with mitochondrial content > 20% were excluded; 4) genes expressed in less than 5 cells were excluded. Filtered cells were retained for the downstream analyses, with on average 1567 genes and 3263 UMIs per cell. Gene Ontology (GO) and Kyoto Encyclopedia of Genes and Genomes (KEGG) analysis were used with the “clusterProfiler” R package v 3.16.1.^[1]^ Cell-cell interaction (CCI) were predicted based on known ligand–receptor pairs by Cellphone DB (v2.1.0) version.^[2]^ Transcription factor network was constructed by pyscenic (v0.11.0) using scRNA expression matrix and transcription factors in AnimalTFDB.

*Clinical bioinformatics analysis + R language*: Data sources for PTSD: The summary-level Genome-wide association study (GWAS) data associated with PTSD were sourced from the Psychiatric Genomics Consortium Posttraumatic Stress Disorder (PGC-PTSD) Working Group.^[3]^ European pedigree data were specifically chosen, encompassing samples from the PGC and UK Biobank, comprising 23,212 cases and 151,447 controls.^[3]^ The determination of lifetime or current PTSD status was established through the utilization of diverse assessment instruments and varying editions of the Diagnostic and Statistical Manual of Mental Disorders (DSM) such as DSM-III-R, DSM-IV, and DSM-V.

*Data sources for brain structure*: GWAS data pertaining to the morphology of the cerebral cortex were acquired from the ENIGMA consortium.^[4]^ The surface area (SA) and thickness (TH) of the cortex were quantified and computed utilizing magnetic resonance imaging (MRI) across the complete cortical surface and 34 specific brain regions within a cohort of 33,992 individuals of European ancestry.^[4]^ Both weighted and unweighted estimates were derived from the entire brain. The delineation of these 34 regions was guided by the Desikan-Killiany atlas, which provides a systematic partitioning of the cortex and demarcates regional boundaries based on the anatomical features of the cerebral gyrus.

*Selection of instrumental variables*: Based on the foundational principles of Mendelian randomization (MR), we initially identified instrumental variables (IVs) associated with PTSD, using a significance threshold of P < 5E−6. Subsequently, we clustered the IVs utilizing data from the European 1000 Genomes Project (clumped R2 < 0.001, window size = 1 Mb). Following this, we standardized the single nucleotide polymorphisms (SNPs) and eliminated palindromic SNPs. Furthermore, we conducted MR Pleiotropy RESidual Sum and Outlier (MRPRESSO) analysis to pinpoint noteworthy SNPs that may demonstrate pleiotropic effects.^[5]^ To gauge the robustness of our MR methodology, we computed the F-statistics. SNPs with F-statistics below 10, indicating inadequate instrument strength, were excluded from consideration.^[6]^ Our MR investigation adhered to the guidelines outlined in the STROBE-MR Statement.^[7]^

*Statistical analyses*: Statistical analyses were performed using three distinct methodologies: Inverse Variance Weighted (IVW), MR Egger, and Weighted Median, with the IVW causal effect estimated under fixed or multiplicative random effects serving as the primary analytical outcome. MR-PRESSO was utilized to identify and address horizontal pleiotropy and eliminate outliers to ensure the attainment of unbiased estimations. Heterogeneity was assessed through Cochran's Q test, while the Leave-One-Out method was employed to pinpoint individual influential Single Nucleotide Polymorphisms (SNPs). The presence of horizontal pleiotropy was ascertained by examining the non-zero intercept in the MR-Egger analysis. To further scrutinize potential pleiotropy, a search of the PhenoScanner database was conducted to sift out SNPs linked to confounding variables. To account for multiple comparisons, Bonferroni correction was applied to adjust the P-values, with statistical significance set at P < 0.05/138 (3.62 E−4) for causal associations. All statistical procedures were carried out utilizing the TwoSampleMR package (version 0.5.8) within the R environment (version 4.3.1).

References

[1] Yu, G., L.G. Wang, Y. Han,Q.Y. He, *Omics,* **2012.** 16(5): p. 284-7.

[2] Efremova, M., M. Vento-Tormo, S.A. Teichmann,R. Vento-Tormo, *Nat Protoc,* **2020.** 15(4): p. 1484-1506.

[3] Nievergelt, C.M., A.X. Maihofer, T. Klengel, E.G. Atkinson, C.Y. Chen, K.W. Choi, J.R.I. Coleman, S. Dalvie, L.E. Duncan, J. Gelernter, D.F. Levey, M.W. Logue, R. Polimanti, A.C. Provost, A. Ratanatharathorn, M.B. Stein, K. Torres, A.E. Aiello, L.M. Almli, A.B. Amstadter, S.B. Andersen, O.A. Andreassen, P.A. Arbisi, A.E. Ashley-Koch, S.B. Austin, E. Avdibegovic, D. Babić, M. Bækvad-Hansen, D.G. Baker, J.C. Beckham, L.J. Bierut, J.I. Bisson, M.P. Boks, E.A. Bolger, A.D. Børglum, B. Bradley, M. Brashear, G. Breen, R.A. Bryant, A.C. Bustamante, J. Bybjerg-Grauholm, J.R. Calabrese, J.M. Caldas-de-Almeida, A.M. Dale, M.J. Daly, N.P. Daskalakis, J. Deckert, D.L. Delahanty, M.F. Dennis, S.G. Disner, K. Domschke, A. Dzubur-Kulenovic, C.R. Erbes, A. Evans, L.A. Farrer, N.C. Feeny, J.D. Flory, D. Forbes, C.E. Franz, S. Galea, M.E. Garrett, B. Gelaye, E. Geuze, C. Gillespie, A.G. Uka, S.D. Gordon, G. Guffanti, R. Hammamieh, S. Harnal, M.A. Hauser, A.C. Heath, S.M.J. Hemmings, D.M. Hougaard, M. Jakovljevic, M. Jett, E.O. Johnson, I. Jones, T. Jovanovic, X.J. Qin, A.G. Junglen, K.I. Karstoft, M.L. Kaufman, R.C. Kessler, A. Khan, N.A. Kimbrel, A.P. King, N. Koen, H.R. Kranzler, W.S. Kremen, B.R. Lawford, L.A.M. Lebois, C.E. Lewis, S.D. Linnstaedt, A. Lori, B. Lugonja, J.J. Luykx, M.J. Lyons, J. Maples-Keller, C. Marmar, A.R. Martin, N.G. Martin, D. Maurer, M.R. Mavissakalian, A. McFarlane, R.E. McGlinchey, K.A. McLaughlin, S.A. McLean, S. McLeay, D. Mehta, W.P. Milberg, M.W. Miller, R.A. Morey, C.P. Morris, O. Mors, P.B. Mortensen, B.M. Neale, E.C. Nelson, M. Nordentoft, S.B. Norman, M. O'Donnell, H.K. Orcutt, M.S. Panizzon, E.S. Peters, A.L. Peterson, M. Peverill, R.H. Pietrzak, M.A. Polusny, J.P. Rice, S. Ripke, V.B. Risbrough, A.L. Roberts, A.O. Rothbaum, B.O. Rothbaum, P. Roy-Byrne, K. Ruggiero, A. Rung, B.P.F. Rutten, N.L. Saccone, S.E. Sanchez, D. Schijven, S. Seedat, A.V. Seligowski, J.S. Seng, C.M. Sheerin, D. Silove, A.K. Smith, J.W. Smoller, S.R. Sponheim, D.J. Stein, J.S. Stevens, J.A. Sumner, M.H. Teicher, W.K. Thompson, E. Trapido, M. Uddin, R.J. Ursano, L.L. van den Heuvel, M. Van Hooff, E. Vermetten, C.H. Vinkers, J. Voisey, Y. Wang, Z. Wang, T. Werge, M.A. Williams, D.E. Williamson, S. Winternitz, C. Wolf, E.J. Wolf, J.D. Wolff, R. Yehuda, R.M. Young, K.A. Young, H. Zhao, L.A. Zoellner, I. Liberzon, K.J. Ressler, M. Haas,K.C. Koenen, *Nat Commun,* **2019.** 10(1): p. 4558.

[4] Grasby, K.L., N. Jahanshad, J.N. Painter, L. Colodro-Conde, J. Bralten, D.P. Hibar, P.A. Lind, F. Pizzagalli, C.R.K. Ching, M.A.B. McMahon, N. Shatokhina, L.C.P. Zsembik, S.I. Thomopoulos, A.H. Zhu, L.T. Strike, I. Agartz, S. Alhusaini, M.A.A. Almeida, D. Alnæs, I.K. Amlien, M. Andersson, T. Ard, N.J. Armstrong, A. Ashley-Koch, J.R. Atkins, M. Bernard, R.M. Brouwer, E.E.L. Buimer, R. Bülow, C. Bürger, D.M. Cannon, M. Chakravarty, Q. Chen, J.W. Cheung, B. Couvy-Duchesne, A.M. Dale, S. Dalvie, T.K. de Araujo, G.I. de Zubicaray, S.M.C. de Zwarte, A. den Braber, N.T. Doan, K. Dohm, S. Ehrlich, H.R. Engelbrecht, S. Erk, C.C. Fan, I.O. Fedko, S.F. Foley, J.M. Ford, M. Fukunaga, M.E. Garrett, T. Ge, S. Giddaluru, A.L. Goldman, M.J. Green, N.A. Groenewold, D. Grotegerd, T.P. Gurholt, B.A. Gutman, N.K. Hansell, M.A. Harris, M.B. Harrison, C.C. Haswell, M. Hauser, S. Herms, D.J. Heslenfeld, N.F. Ho, D. Hoehn, P. Hoffmann, L. Holleran, M. Hoogman, J.J. Hottenga, M. Ikeda, D. Janowitz, I.E. Jansen, T. Jia, C. Jockwitz, R. Kanai, S. Karama, D. Kasperaviciute, T. Kaufmann, S. Kelly, M. Kikuchi, M. Klein, M. Knapp, A.R. Knodt, B. Krämer, M. Lam, T.M. Lancaster, P.H. Lee, T.A. Lett, L.B. Lewis, I. Lopes-Cendes, M. Luciano, F. Macciardi, A.F. Marquand, S.R. Mathias, T.R. Melzer, Y. Milaneschi, N. Mirza-Schreiber, J.C.V. Moreira, T.W. Mühleisen, B. Müller-Myhsok, P. Najt, S. Nakahara, K. Nho, L.M. Olde Loohuis, D.P. Orfanos, J.F. Pearson, T.L. Pitcher, B. Pütz, Y. Quidé, A. Ragothaman, F.M. Rashid, W.R. Reay, R. Redlich, C.S. Reinbold, J. Repple, G. Richard, B.C. Riedel, S.L. Risacher, C.S. Rocha, N.R. Mota, L. Salminen, A. Saremi, A.J. Saykin, F. Schlag, L. Schmaal, P.R. Schofield, R. Secolin, C.Y. Shapland, L. Shen, J. Shin, E. Shumskaya, I.E. Sønderby, E. Sprooten, K.E. Tansey, A. Teumer, A. Thalamuthu, D. Tordesillas-Gutiérrez, J.A. Turner, A. Uhlmann, C.L. Vallerga, D. van der Meer, M.M.J. van Donkelaar, L. van Eijk, T.G.M. van Erp, N.E.M. van Haren, D. van Rooij, M.J. van Tol, J.H. Veldink, E. Verhoef, E. Walton, M. Wang, Y. Wang, J.M. Wardlaw, W. Wen, L.T. Westlye, C.D. Whelan, S.H. Witt, K. Wittfeld, C. Wolf, T. Wolfers, J.Q. Wu, C.L. Yasuda, D. Zaremba, Z. Zhang, M.P. Zwiers, E. Artiges, A.A. Assareh, R. Ayesa-Arriola, A. Belger, C.L. Brandt, G.G. Brown, S. Cichon, J.E. Curran, G.E. Davies, F. Degenhardt, M.F. Dennis, B. Dietsche, S. Djurovic, C.P. Doherty, R. Espiritu, D. Garijo, Y. Gil, P.A. Gowland, R.C. Green, A.N. Häusler, W. Heindel, B.C. Ho, W.U. Hoffmann, F. Holsboer, G. Homuth, N. Hosten, C.R. Jack, Jr., M. Jang, A. Jansen, N.A. Kimbrel, K. Kolskår, S. Koops, A. Krug, K.O. Lim, J.J. Luykx, D.H. Mathalon, K.A. Mather, V.S. Mattay, S. Matthews, J. Mayoral Van Son, S.C. McEwen, I. Melle, D.W. Morris, B.A. Mueller, M. Nauck, J.E. Nordvik, M.M. Nöthen, D.S. O'Leary, N. Opel, M.P. Martinot, G.B. Pike, A. Preda, E.B. Quinlan, P.E. Rasser, V. Ratnakar, S. Reppermund, V.M. Steen, P.A. Tooney, F.R. Torres, D.J. Veltman, J.T. Voyvodic, R. Whelan, T. White, H. Yamamori, H.H.H. Adams, J.C. Bis, S. Debette, C. Decarli, M. Fornage, V. Gudnason, E. Hofer, M.A. Ikram, L. Launer, W.T. Longstreth, O.L. Lopez, B. Mazoyer, T.H. Mosley, G.V. Roshchupkin, C.L. Satizabal, R. Schmidt, S. Seshadri, Q. Yang, M.K.M. Alvim, D. Ames, T.J. Anderson, O.A. Andreassen, A. Arias-Vasquez, M.E. Bastin, B.T. Baune, J.C. Beckham, J. Blangero, D.I. Boomsma, H. Brodaty, H.G. Brunner, R.L. Buckner, J.K. Buitelaar, J.R. Bustillo, W. Cahn, M.J. Cairns, V. Calhoun, V.J. Carr, X. Caseras, S. Caspers, G.L. Cavalleri, F. Cendes, A. Corvin, B. Crespo-Facorro, J.C. Dalrymple-Alford, U. Dannlowski, E.J.C. de Geus, I.J. Deary, N. Delanty, C. Depondt, S. Desrivières, G. Donohoe, T. Espeseth, G. Fernández, S.E. Fisher, H. Flor, A.J. Forstner, C. Francks, B. Franke, D.C. Glahn, R.L. Gollub, H.J. Grabe, O. Gruber, A.K. Håberg, A.R. Hariri, C.A. Hartman, R. Hashimoto, A. Heinz, F.A. Henskens, M.H.J. Hillegers, P.J. Hoekstra, A.J. Holmes, L.E. Hong, W.D. Hopkins, H.E. Hulshoff Pol, T.L. Jernigan, E.G. Jönsson, R.S. Kahn, M.A. Kennedy, T.T.J. Kircher, P. Kochunov, J.B.J. Kwok, S. Le Hellard, C.M. Loughland, N.G. Martin, J.L. Martinot, C. McDonald, K.L. McMahon, A. Meyer-Lindenberg, P.T. Michie, R.A. Morey, B. Mowry, L. Nyberg, J. Oosterlaan, R.A. Ophoff, C. Pantelis, T. Paus, Z. Pausova, B. Penninx, T.J.C. Polderman, D. Posthuma, M. Rietschel, J.L. Roffman, L.M. Rowland, P.S. Sachdev, P.G. Sämann, U. Schall, G. Schumann, R.J. Scott, K. Sim, S.M. Sisodiya, J.W. Smoller, I.E. Sommer, B. St Pourcain, D.J. Stein, A.W. Toga, J.N. Trollor, N.J.A. Van der Wee, D. van 't Ent, H. Völzke, H. Walter, B. Weber, D.R. Weinberger, M.J. Wright, J. Zhou, J.L. Stein, P.M. Thompson,S.E. Medland, *Science,* **2020.** 367(6484).

[5] Chen, X., J. Kong, J. Pan, K. Huang, W. Zhou, X. Diao, J. Cai, J. Zheng, X. Yang, W. Xie, H. Yu, J. Li, L. Pei, W. Dong, H. Qin, J. Huang,T. Lin, *EBioMedicine,* **2021.** 72: p. 103592.

[6] Burgess, S.,S.G. Thompson, *Int J Epidemiol,* **2011.** 40(3): p. 755-64.

[7] Skrivankova, V.W., R.C. Richmond, B.A.R. Woolf, J. Yarmolinsky, N.M. Davies, S.A. Swanson, T.J. VanderWeele, J.P.T. Higgins, N.J. Timpson, N. Dimou, C. Langenberg, R.M. Golub, E.W. Loder, V. Gallo, A. Tybjaerg-Hansen, G. Davey Smith, M. Egger,J.B. Richards, *Jama,* **2021.** 326(16): p. 1614-1621.

**Table S1. Statistical comparison and analysis data between groups for the results of Figure 1H and 1I**

| Tukey's multiple comparisons test | Mean Diff. | 95.00% CI of diff. | Summary | Adjusted P Value |
| --- | --- | --- | --- | --- |
| Central/Total distance (%) | | | | |
| 7 Days |  |  |  |  |
| PTSD vs. TBI-5.5Mpa | 33.4 | 24.83 to 41.97 | **** | <0.0001 |
| PTSD vs. TBI+PTSD-5.5Mpa | 36.4 | 27.83 to 44.97 | **** | <0.0001 |
| PTSD vs. TBI-4.5Mpa | 9.4 | 0.8258 to 17.97 | * | 0.021 |
| PTSD vs. TBI+PTSD-4.5Mpa | 11.2 | 2.626 to 19.77 | ** | 0.0024 |
| PTSD vs. TBI-3.5Mpa | 1.6 | -6.974 to 10.17 | ns | 0.9991 |
| PTSD vs. TBI+PTSD-3.5Mpa | 4.2 | -4.374 to 12.77 | ns | 0.801 |
| 14 Days |  |  |  |  |
| PTSD vs. TBI-5.5Mpa | 21.4 | 12.83 to 29.97 | **** | <0.0001 |
| PTSD vs. TBI+PTSD-5.5Mpa | 21.2 | 12.63 to 29.77 | **** | <0.0001 |
| PTSD vs. TBI-4.5Mpa | 8.8 | 0.2258 to 17.37 | * | 0.0398 |
| PTSD vs. TBI+PTSD-4.5Mpa | 15.4 | 6.826 to 23.97 | **** | <0.0001 |
| PTSD vs. TBI-3.5Mpa | -16.4 | -24.97 to -7.826 | **** | <0.0001 |
| PTSD vs. TBI+PTSD-3.5Mpa | 1.2 | -7.374 to 9.774 | ns | 0.9999 |
| 21 Days |  |  |  |  |
| PTSD vs. TBI-5.5Mpa | 21.46 | 12.89 to 30.03 | **** | <0.0001 |
| PTSD vs. TBI+PTSD-5.5Mpa | 19.94 | 11.37 to 28.51 | **** | <0.0001 |
| PTSD vs. TBI-4.5Mpa | 12.74 | 4.166 to 21.31 | *** | 0.0003 |
| PTSD vs. TBI+PTSD-4.5Mpa | 15.42 | 6.846 to 23.99 | **** | <0.0001 |
| PTSD vs. TBI-3.5Mpa | -12.38 | -20.95 to -3.806 | *** | 0.0005 |
| PTSD vs. TBI+PTSD-3.5Mpa | 5.3 | -3.274 to 13.87 | ns | 0.5502 |
| 28 Days |  |  |  |  |
| PTSD vs. TBI-5.5Mpa | 42.14 | 33.57 to 50.71 | **** | <0.0001 |
| PTSD vs. TBI+PTSD-5.5Mpa | 38.44 | 29.87 to 47.01 | **** | <0.0001 |
| PTSD vs. TBI-4.5Mpa | 14.84 | 6.266 to 23.41 | **** | <0.0001 |
| PTSD vs. TBI+PTSD-4.5Mpa | 25.3 | 16.73 to 33.87 | **** | <0.0001 |
| PTSD vs. TBI-3.5Mpa | 9 | 0.4258 to 17.57 | * | 0.0324 |
| PTSD vs. TBI+PTSD-3.5Mpa | 1.86 | -6.714 to 10.43 | ns | 0.9977 |
| Central/Total time (%) | | | | |
| 7 Days |  |  |  |  |
| PTSD vs. TBI-5.5Mpa | 13.8 | 10.25 to 17.35 | **** | <0.0001 |
| PTSD vs. TBI+PTSD-5.5Mpa | 12 | 8.447 to 15.55 | **** | <0.0001 |
| PTSD vs. TBI-4.5Mpa | -0.2 | -3.753 to 3.353 | ns | >0.9999 |
| PTSD vs. TBI+PTSD-4.5Mpa | 1.4 | -2.153 to 4.953 | ns | 0.9263 |
| PTSD vs. TBI-3.5Mpa | 0.8 | -2.753 to 4.353 | ns | 0.997 |
| PTSD vs. TBI+PTSD-3.5Mpa | 3.2 | -0.3527 to 6.753 | ns | 0.1104 |
| TBI-5.5Mpa vs. TBI+PTSD-5.5Mpa | -1.8 | -5.353 to 1.753 | ns | 0.7721 |
| 14 Days |  |  |  |  |
| PTSD vs. TBI-5.5Mpa | 7.94 | 4.387 to 11.49 | **** | <0.0001 |
| PTSD vs. TBI+PTSD-5.5Mpa | 9.26 | 5.707 to 12.81 | **** | <0.0001 |
| PTSD vs. TBI-4.5Mpa | 2.6 | -0.9527 to 6.153 | ns | 0.3266 |
| PTSD vs. TBI+PTSD-4.5Mpa | 5.6 | 2.047 to 9.153 | **** | <0.0001 |
| PTSD vs. TBI-3.5Mpa | -6.6 | -10.15 to -3.047 | **** | <0.0001 |
| PTSD vs. TBI+PTSD-3.5Mpa | 0.4 | -3.153 to 3.953 | ns | >0.9999 |
| 21 Days |  |  |  |  |
| PTSD vs. TBI-5.5Mpa | 7.1 | 3.547 to 10.65 | **** | <0.0001 |
| PTSD vs. TBI+PTSD-5.5Mpa | 7.52 | 3.967 to 11.07 | **** | <0.0001 |
| PTSD vs. TBI-4.5Mpa | 2.5 | -1.053 to 6.053 | ns | 0.3777 |
| PTSD vs. TBI+PTSD-4.5Mpa | 5.2 | 1.647 to 8.753 | *** | 0.0004 |
| PTSD vs. TBI-3.5Mpa | -6.12 | -9.673 to -2.567 | **** | <0.0001 |
| PTSD vs. TBI+PTSD-3.5Mpa | 0 | -3.553 to 3.553 | ns | >0.9999 |
| 28 Days |  |  |  |  |
| PTSD vs. TBI-5.5Mpa | 14.94 | 11.39 to 18.49 | **** | <0.0001 |
| PTSD vs. TBI+PTSD-5.5Mpa | 15.22 | 11.67 to 18.77 | **** | <0.0001 |
| PTSD vs. TBI-4.5Mpa | 3.16 | -0.3927 to 6.713 | ns | 0.12 |
| PTSD vs. TBI+PTSD-4.5Mpa | 5.54 | 1.987 to 9.093 | *** | 0.0001 |
| PTSD vs. TBI-3.5Mpa | 0.22 | -3.333 to 3.773 | ns | >0.9999 |
| PTSD vs. TBI+PTSD-3.5Mpa | 3.8 | 0.2473 to 7.353 | * | 0.027 |
| TBI-5.5Mpa vs. TBI+PTSD-5.5Mpa | 0.28 | -3.273 to 3.833 | ns | >0.9999 |
| Open arms/Total distance (%) | | | | |
| 7 Days |  |  |  |  |
| PTSD vs. TBI-5.5Mpa | 12.78 | 10.58 to 14.98 | **** | <0.0001 |
| PTSD vs. TBI+PTSD-5.5Mpa | 11.8 | 9.599 to 14.00 | **** | <0.0001 |
| PTSD vs. TBI-4.5Mpa | -1.4 | -3.601 to 0.8011 | ns | 0.5128 |
| PTSD vs. TBI+PTSD-4.5Mpa | 0.2 | -2.001 to 2.401 | ns | >0.9999 |
| PTSD vs. TBI-3.5Mpa | -1.2 | -3.401 to 1.001 | ns | 0.7 |
| PTSD vs. TBI+PTSD-3.5Mpa | -1.4 | -3.601 to 0.8011 | ns | 0.5128 |
| 14 Days |  |  |  |  |
| PTSD vs. TBI-5.5Mpa | 8.18 | 5.979 to 10.38 | **** | <0.0001 |
| PTSD vs. TBI+PTSD-5.5Mpa | 7 | 4.799 to 9.201 | **** | <0.0001 |
| PTSD vs. TBI-4.5Mpa | 3 | 0.7989 to 5.201 | ** | 0.0013 |
| PTSD vs. TBI+PTSD-4.5Mpa | 4.4 | 2.199 to 6.601 | **** | <0.0001 |
| PTSD vs. TBI-3.5Mpa | -7 | -9.201 to -4.799 | **** | <0.0001 |
| PTSD vs. TBI+PTSD-3.5Mpa | 1.2 | -1.001 to 3.401 | ns | 0.7 |
| 21 Days |  |  |  |  |
| PTSD vs. TBI-5.5Mpa | 6.7 | 4.499 to 8.901 | **** | <0.0001 |
| PTSD vs. TBI+PTSD-5.5Mpa | 6.2 | 3.999 to 8.401 | **** | <0.0001 |
| PTSD vs. TBI-4.5Mpa | 0.62 | -1.581 to 2.821 | ns | 0.9883 |
| PTSD vs. TBI+PTSD-4.5Mpa | 4.24 | 2.039 to 6.441 | **** | <0.0001 |
| PTSD vs. TBI-3.5Mpa | -7.18 | -9.381 to -4.979 | **** | <0.0001 |
| PTSD vs. TBI+PTSD-3.5Mpa | 0.7 | -1.501 to 2.901 | ns | 0.9765 |
| 28 Days |  |  |  |  |
| PTSD vs. TBI-5.5Mpa | 11.94 | 9.739 to 14.14 | **** | <0.0001 |
| PTSD vs. TBI+PTSD-5.5Mpa | 13.52 | 11.32 to 15.72 | **** | <0.0001 |
| PTSD vs. TBI-4.5Mpa | 5.48 | 3.279 to 7.681 | **** | <0.0001 |
| PTSD vs. TBI+PTSD-4.5Mpa | 8.42 | 6.219 to 10.62 | **** | <0.0001 |
| PTSD vs. TBI-3.5Mpa | -1.72 | -3.921 to 0.4811 | ns | 0.2465 |
| PTSD vs. TBI+PTSD-3.5Mpa | 1.48 | -0.7211 to 3.681 | ns | 0.4385 |
| Open arms/Total time (%) | | | | |
| 7 Days |  |  |  |  |
| PTSD vs. TBI-5.5Mpa | 22.64 | 20.49 to 24.79 | **** | <0.0001 |
| PTSD vs. TBI+PTSD-5.5Mpa | 23.34 | 21.19 to 25.49 | **** | <0.0001 |
| PTSD vs. TBI-4.5Mpa | 1.2 | -0.9546 to 3.355 | ns | 0.6767 |
| PTSD vs. TBI+PTSD-4.5Mpa | 4.6 | 2.445 to 6.755 | **** | <0.0001 |
| PTSD vs. TBI-3.5Mpa | 1.8 | -0.3546 to 3.955 | ns | 0.175 |
| PTSD vs. TBI+PTSD-3.5Mpa | 2 | -0.1546 to 4.155 | ns | 0.0896 |
| 14 Days |  |  |  |  |
| PTSD vs. TBI-5.5Mpa | 13.26 | 11.11 to 15.41 | **** | <0.0001 |
| PTSD vs. TBI+PTSD-5.5Mpa | 13.08 | 10.93 to 15.23 | **** | <0.0001 |
| PTSD vs. TBI-4.5Mpa | 2.2 | 0.04543 to 4.355 | * | 0.0417 |
| PTSD vs. TBI+PTSD-4.5Mpa | 8.2 | 6.045 to 10.35 | **** | <0.0001 |
| PTSD vs. TBI-3.5Mpa | -7.6 | -9.755 to -5.445 | **** | <0.0001 |
| PTSD vs. TBI+PTSD-3.5Mpa | 0.2 | -1.955 to 2.355 | ns | >0.9999 |
| 21 Days |  |  |  |  |
| PTSD vs. TBI-5.5Mpa | 8.38 | 6.225 to 10.53 | **** | <0.0001 |
| PTSD vs. TBI+PTSD-5.5Mpa | 8.08 | 5.925 to 10.23 | **** | <0.0001 |
| PTSD vs. TBI-4.5Mpa | 3.36 | 1.205 to 5.515 | *** | 0.0001 |
| PTSD vs. TBI+PTSD-4.5Mpa | 8.3 | 6.145 to 10.45 | **** | <0.0001 |
| PTSD vs. TBI-3.5Mpa | -12.14 | -14.29 to -9.985 | **** | <0.0001 |
| PTSD vs. TBI+PTSD-3.5Mpa | -1.3 | -3.455 to 0.8546 | ns | 0.5811 |
| 28 Days |  |  |  |  |
| PTSD vs. TBI-5.5Mpa | 21.1 | 18.95 to 23.25 | **** | <0.0001 |
| PTSD vs. TBI+PTSD-5.5Mpa | 20.9 | 18.75 to 23.05 | **** | <0.0001 |
| PTSD vs. TBI-4.5Mpa | 6.28 | 4.125 to 8.435 | **** | <0.0001 |
| PTSD vs. TBI+PTSD-4.5Mpa | 12.36 | 10.21 to 14.51 | **** | <0.0001 |
| PTSD vs. TBI-3.5Mpa | -0.22 | -2.375 to 1.935 | ns | >0.9999 |
| PTSD vs. TBI+PTSD-3.5Mpa | 0.58 | -1.575 to 2.735 | ns | 0.9911 |

**Table S2. Detailed information about the antibodies and kits**

| Antibodies and Kits | Brand | Article number |
| --- | --- | --- |
| Tunel Kit | Beyotime | C1089 |
| Annexin-FITC/PI Kit | Beyotime | C1062 |
| IBa-1 | Abcam | [ab178846](https://www.abcam.cn/products/primary-antibodies/iba1-antibody-epr16588-ab178846.html) |
| DAPI | Abcam | ab104139 |
| NeuN | Abcam | ab177487 |
| MAP2 | Abcam | ab183830 |
| Smad3 | Abcam | ab208182 |
| Gabra6 | Abcam | ab300069 |
| β-actin | Abcam | ab8226 |
| Smad3(S423+S425) | Abcam | ab52903 |
| H3 | Abcam | ab1791 |
| Smad4 | Abcam | ab230815 |
| Homer1 | Abcam | ab184955 |
| PSD-95 | Abcam | ab238135 |
| PKA-RII | Abcam | ab32514 |
| PKA-RII(S99) | Abcam | ab32390 |
| PKA-C | CST | 4782 |
| PKA-C(T197) | CST | 5661 |
| CREB | CST | 9197 |
| CREB(S133) | CST | 9198 |
| Gabra6(15-26aa) | Thermo | PA5-142768 |
| Gabra6(66-121aa) | Thermo | PA5-36590 |
| Gabra6(43-242aa) | Thermo | PA5-119957 |
| Smad3 | MCE | HY-P73636 |

**Table S3. Primers for qPCR**

| Primers | Primer sequence |
| --- | --- |
| IL-1β: F | 5'-GAA ATG CCA CCT TTT GAC AGT G-3' |
| IL-1β: R | 5'-TGG ATG CTC TCA TCA GGA CAG-3' |
| TNF-α: F | 5'-GAC GTG GAA CTG GCA GAA GAG-3' |
| TNF-α: R | 5'-TTG GTG GTT TGT GAG TGT GAG-3' |
| IL-10: F | 5'-GCT CTT ACT GAC TGG CAT GAG-3' |
| IL-10: R | 5'-CGC AGC TCT AGG AGC ATG TG-3' |
| ERCC6L2: F | 5'-ACA TGG TTG AAA GGG GAA CCT-3' |
| ERCC6L2: R | 5'-GAA GCC TGG AGT TCT ACT GGT-3' |
| SLC6A4: F | 5'-TAT CCA ATG GGT ACT CCG CAG-3' |
| SLC6A4: R | 5'-CCG TTC CCC TTG GTG AAT CT-3' |
| GAD1: F | 5'-CAC AGG TCA CCC TCG ATT TTT-3' |
| GAD1: R | 5'-ACC ATC CAA CGA TCT CTC TCA TC-3' |
| β-actin: F | 5'-GTG ACG TTG ACA TCC GTA AAG A-3' |
| β-actin: R | 5'-GCC GGA CTC ATC GTA CTC C-3' |
| PKA-RII: F | 5'-GAG GAG GAT AAC GAT CCA AGG G-3' |
| PKA-RII: R | 5'-TGC TCG TCA GTT TTG ACA ATC TT-3' |
| PKA-C: F | 5'-TCC AGC TCC AAC GAT GTG AAA-3' |
| PKA-C: R | 5'-ACT GAT CCA ACT GGG CTG TAT T-3' |
| CREB: F | 5'-AGC AGC TCA TGC AAC ATC ATC-3' |
| CREB: R | 5'-AGT CCT TAC AGG AAG ACT GAA CT-3' |


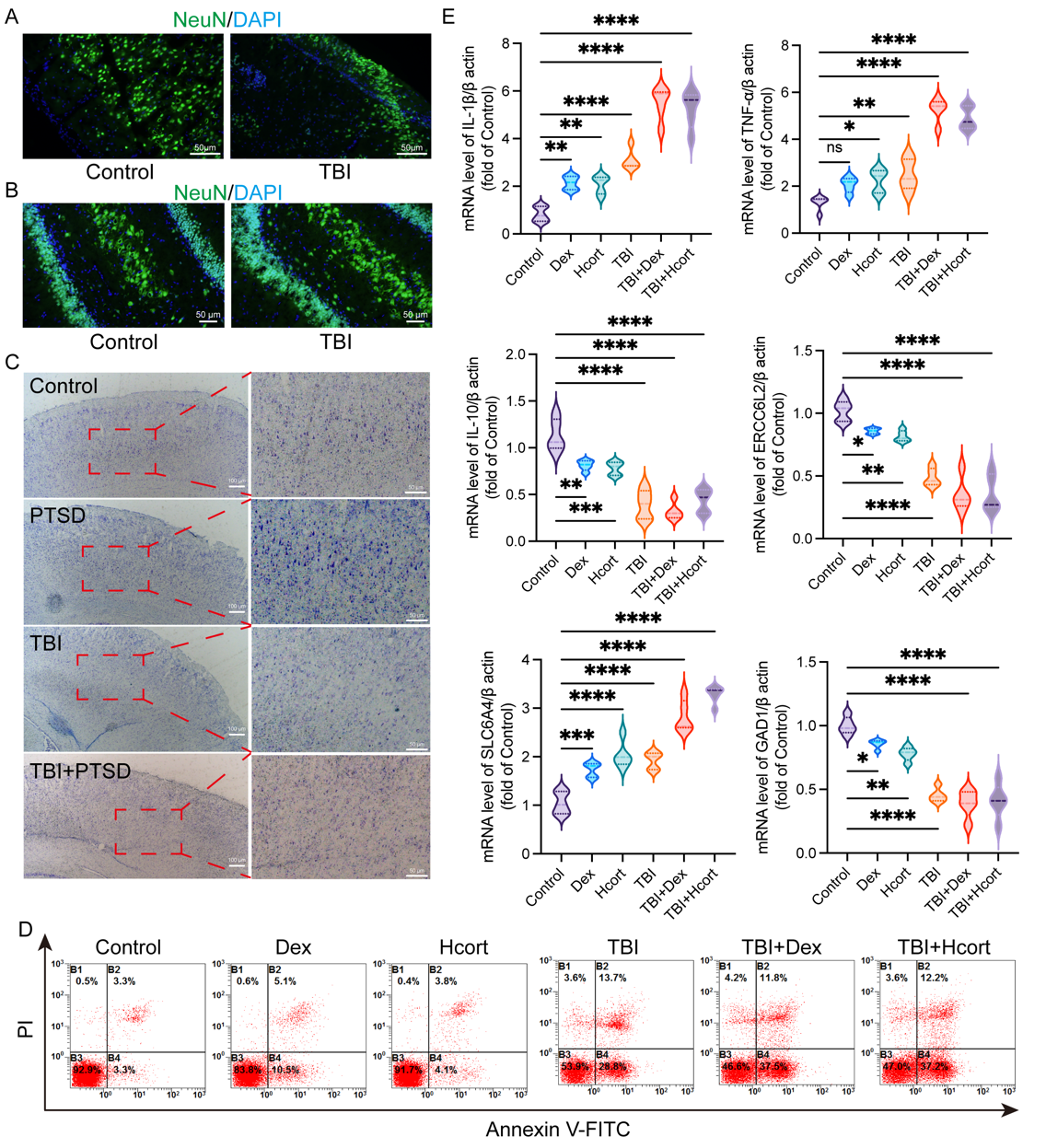


**Figure S1.** Pathological examination and PTSD-related parameters of model mice.

A) Immunofluorescent staining of cortical neurons after 2 weeks of TBI. B) Immunofluorescent staining of hippocampal neurons after 2 weeks of TBI. C) The Nissl staining of cortical neurons in different group after 2 weeks of TBI. D) Flow cytometry was used to detect the apoptotic rate of primary neurons in each group in vitro. E) mRNA levels of IL-1β, TNF-α, SLC6A4, IL-10, ERCC6L2, and GAD1 in different modified primary neurons. IL-1β: F (5, 24) = 66.11, *P < 0.0001*; TNF-α: F (5, 24) = 58.68, *P < 0.0001*; IL-10: F (5, 24) = 35.26, *P < 0.0001*; ERCC6L2: F (5, 24) = 46.47, *P < 0.0001*; SLC6A4: F (5, 24) = 66.08, *P < 0.0001*; GAD1: F (5, 24) = 50.49, *P < 0.0001*; The data were analyzed using one-way ANOVA (**E**), and all data are expressed as the mean ± standard deviation. **P < 0.05*, ***P < 0.01*, ****P < 0.001* and *****P < 0.0001* represents a statistically significant difference between the two groups. ns, not significant. Each experiment was repeated three times.


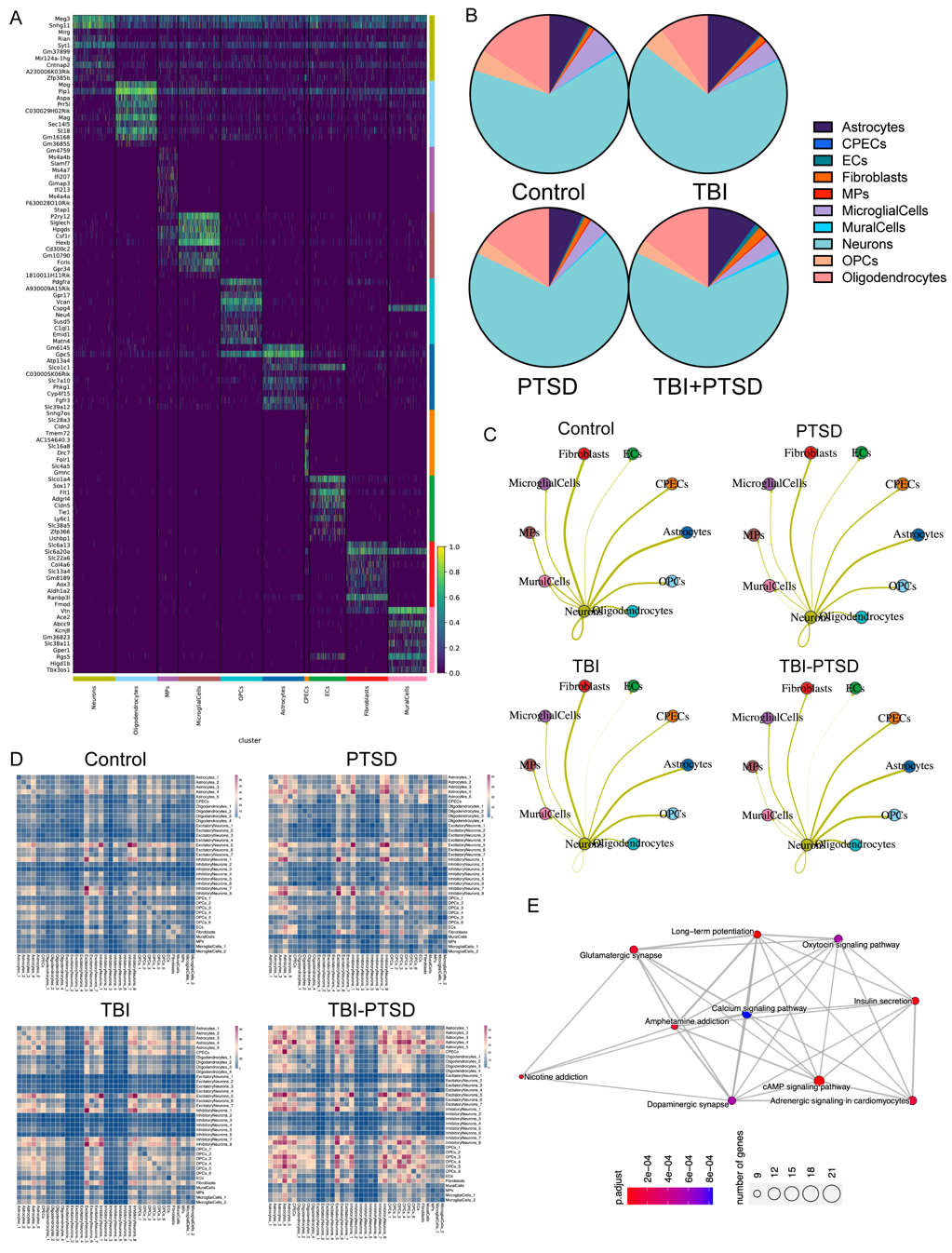


**Figure S2.** Cellular interactions between groups.

A) Top 10 markers for each cell type. B) The proportion of various types of cells. C) Shell diagram showing cell-cell interaction between groups. D) Heat map showing cell-cell interaction between groups. E) KEGG analysis of Gabra6^+^ neurons in the cortex of mice in the PTSD group and TBI-induced PTSD groups.


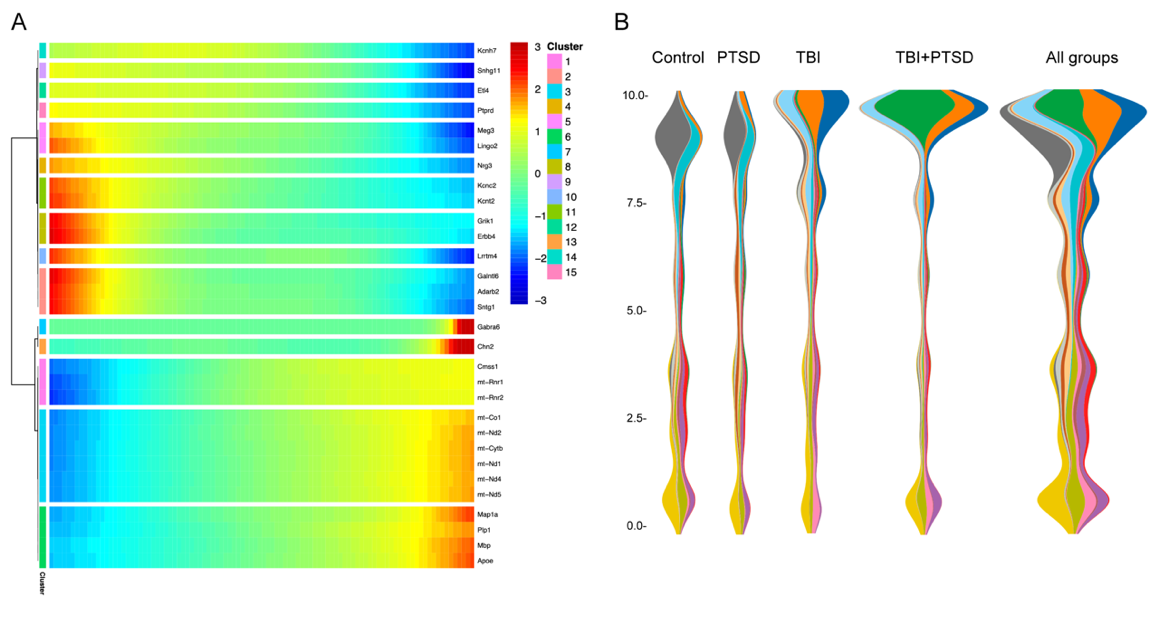


**Figure S3.** Pseudotime analysis of snRNA-seq results.

A) Pseudotime analysis of gene profile expression. B) Pseudotime analysis of cell subtypes in each group.


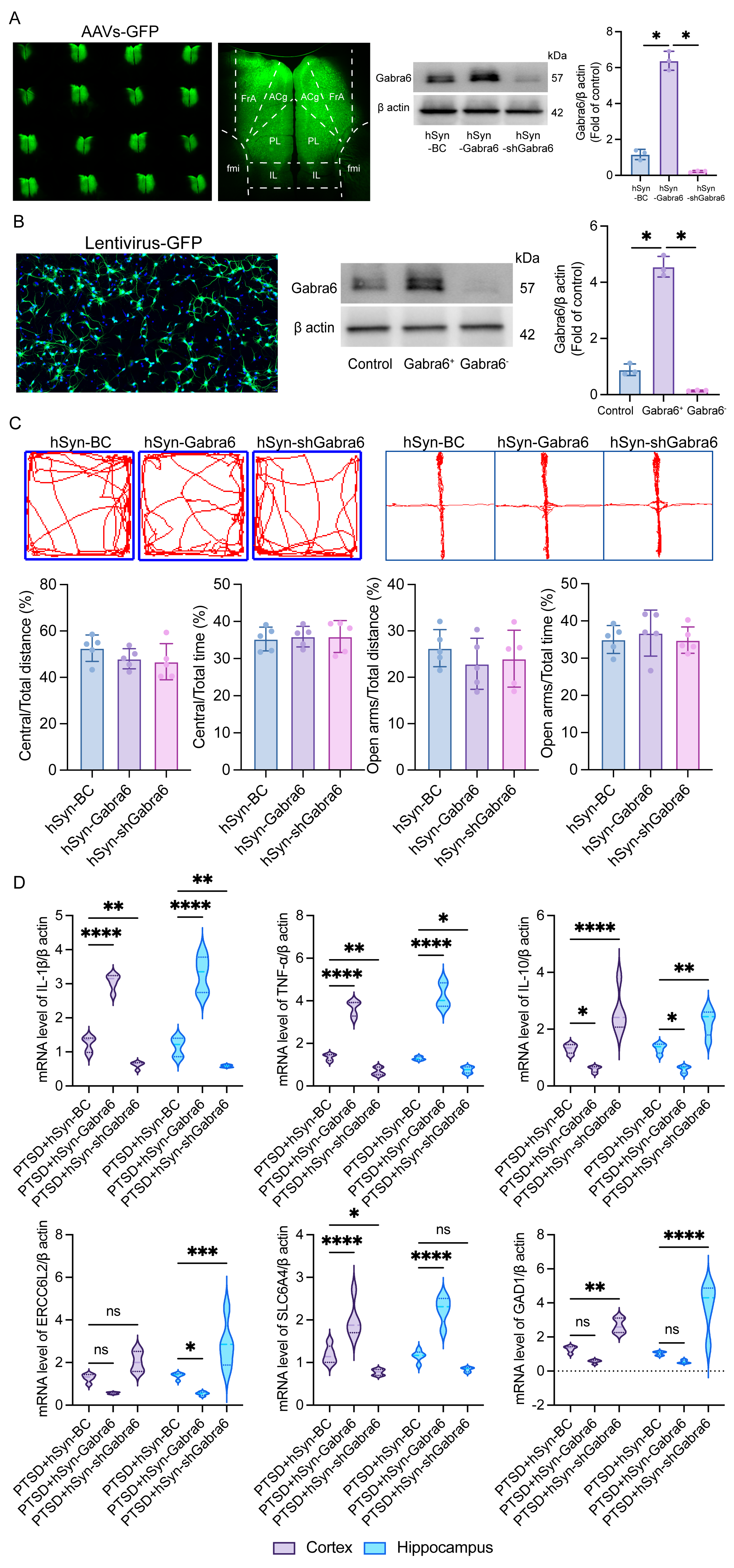


**Figure S4.** The expression of PTSD-related parameters in the cortex and hippocampus.

A) Infection efficiency and interference efficiency of AAVs in vivo (F (2, 6) = 270.9, *P* < *0.0001*). B) The infection efficiency and interference efficiency of lentivirus in primary neurons (F (2, 6) = 282.20, *P* < *0.0001*). C) The effect of different expression of Gabra6 in the prefrontal cortex of mice on behavior. Central/Total distance: F (2, 12) = 1.262, *P = 0.3182*; Central/Total time: F (2, 12) = 0.05651, *P = 0.9453*; Open arms/Total distance: F (2, 12) = 0.5276, *P = 0.6031*; Open arms/Total time: F (2, 12) = 0.2563, *P = 0.7781*; D) mRNA levels of IL-1β, TNF-α, SLC6A4, IL-10, ERCC6L2, and GAD1 in different modified primary neurons. IL-1β: F_cort_ (2, 12) = 207.1, *P < 0.0001*; F_hippo_ (2, 12) = 84.17, *P < 0.0001*; TNF-α: F_cort_ (2, 12) = 194.8, *P < 0.0001*; F_hippo_ (2, 12) = 148.4, *P < 0.0001*; IL-10: F_cort_ (2, 12) = 26.37, *P < 0.0001*; F_hippo_ (2, 12) = 40.71, *P < 0.0001*; ERCC6L2: F_cort_ (2, 12) = 30.78, *P < 0.0001*; F_hippo_ (2, 12) = 15.69, *P = 0.0004*; SLC6A4: F_cort_ (2, 12) = 25.79, *P < 0.0001*; F_hippo_ (2, 12) = 68.46, *P < 0.0001*; GAD1: F_cort_ (2, 12) = 75.66, *P < 0.0001*; F_hippo_ (2, 12) = 23.50, *P < 0.0001*; The data were analyzed using one-way ANOVA, and all data are expressed as the mean ± standard deviation. **P < 0.05*, ***P < 0.01*, ****P < 0.001* and *****P < 0.0001* represents a statistically significant difference between the two groups. ns, not significant. Each experiment was repeated three times.


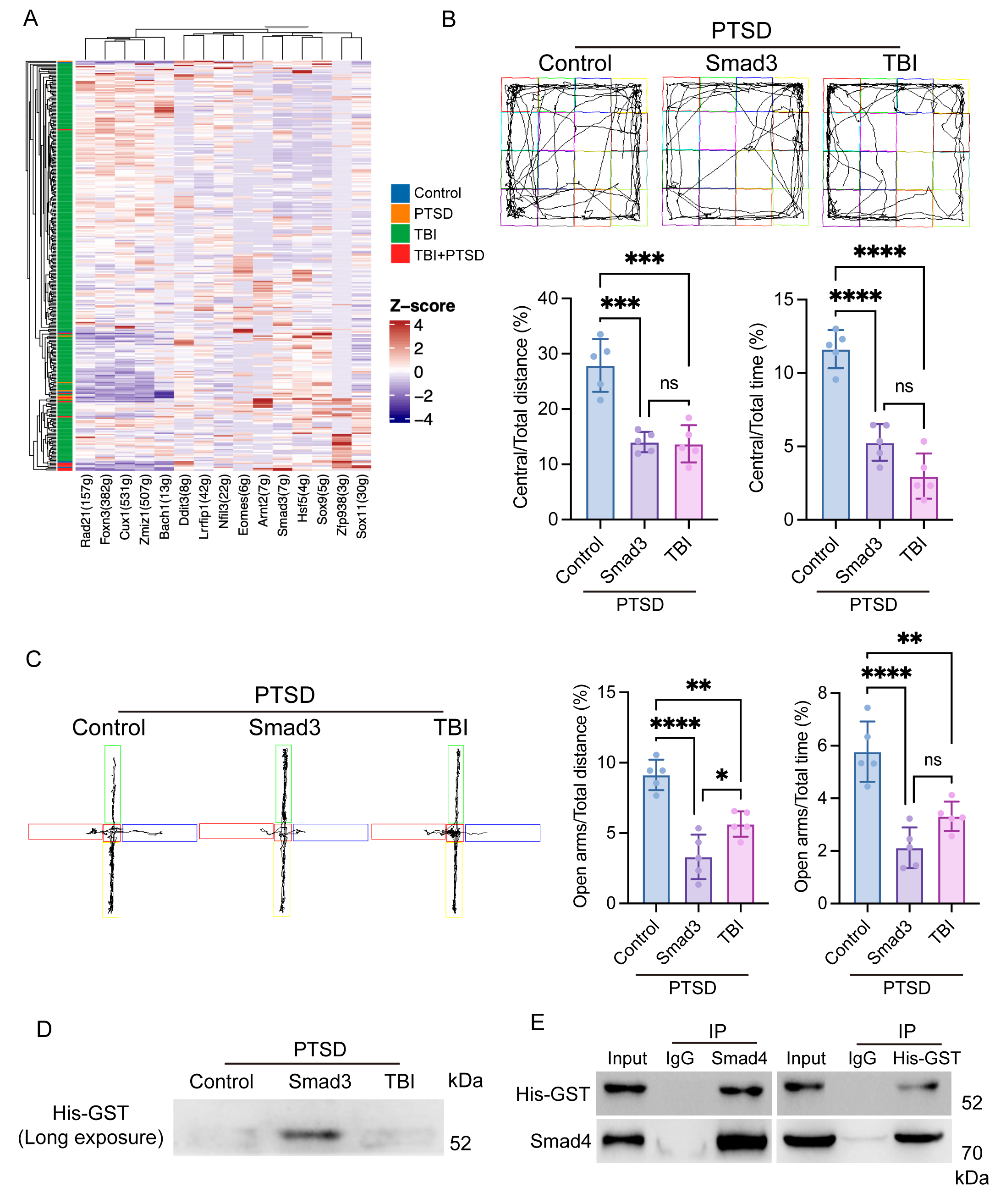


**Figure S5.** Smad3 protein injection promotes PTSD formation in mice.

A) Heat map showing the activation level of patent factors and the number of transcription factors in each group. B) Representative trajectory plots for open field experiments; different modified mice after 2 W after PTSD modeling. Central/Total distance: F (2, 12) = 26.12, *P < 0.0001*; Central/Total time: F (2, 12) = 53.91, *P < 0.0001*; C) Representative trajectory plots for elevated plus maze; different modified mice after 2 W after PTSD modeling. Open arms/Total distance: F (2, 12) = 28.66, *P < 0.0001*; Open arms/Total time: F (2, 12) = 23.48, *P < 0.0001*; D) WB was used to detect the expression levels of exogenous His tags in the prefrontal cortex of mice in each group 2 weeks after modeling. E) The IP experiment was used to detect the binding ability of exogenous Smad3-His-GST protein and endogenous Smad4 protein. The data were analyzed using one-way ANOVA (**B** and **C**), and all data are expressed as the mean ± standard deviation. **P < 0.05*, ***P < 0.01*, ****P < 0.001* and *****P < 0.0001* represents a statistically significant difference between the two groups. Each experiment was repeated three times.


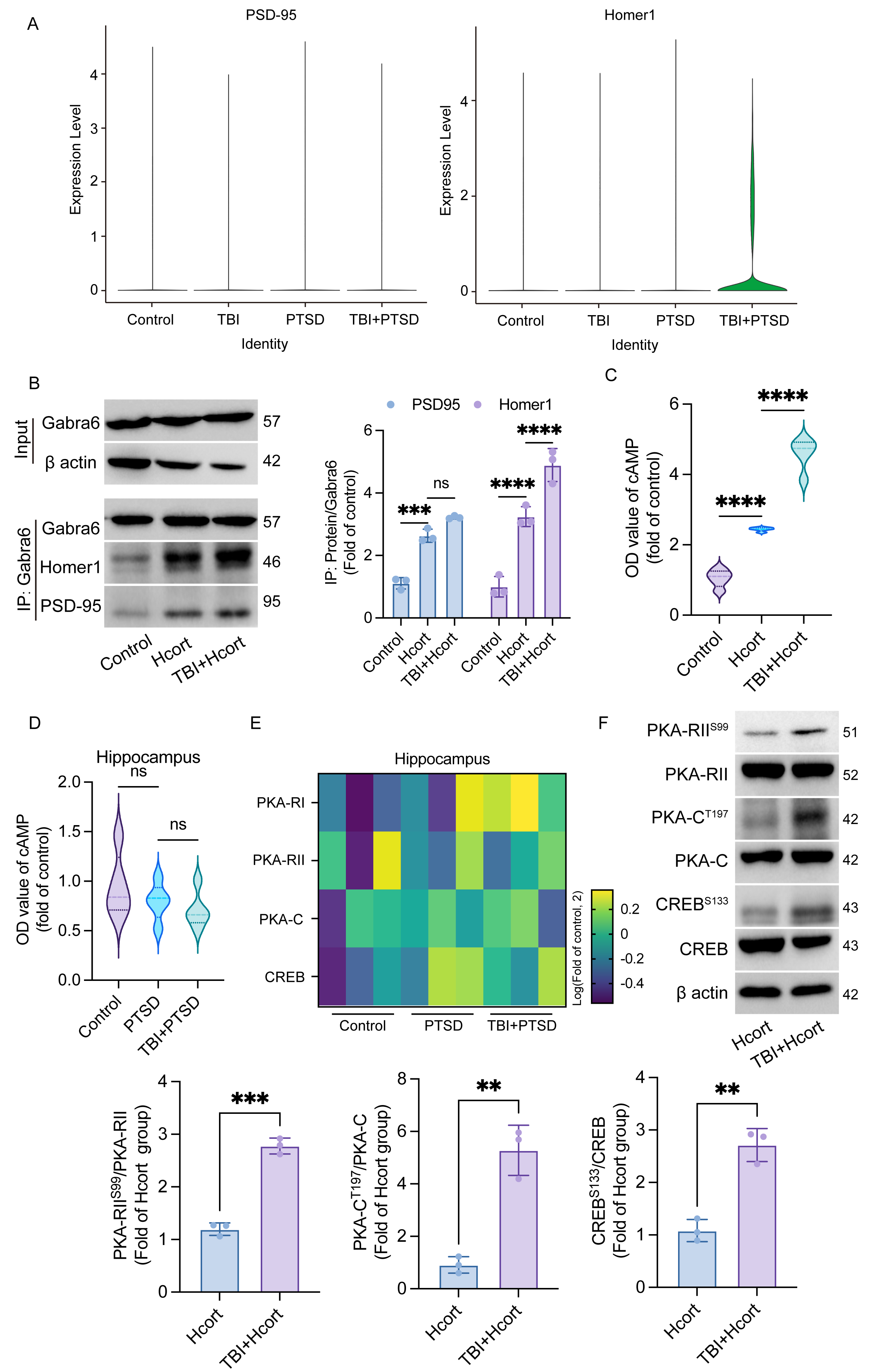


**Figure S6.** Gabra6 binds to Homer1 and PSD-95 and regulates cAMP signaling pathways.

A) snRNA-seq data was analyzed to clarify the expression of Homer1 and PSD-95 in each group. B) Detection of differences in binding ability of proteins in vitro model of each group. PSD95: F (2, 6) = 133.6, *P < 0.0001*; Homer1: F (2, 6) = 70.38, *P < 0.0001*. C) Elisa was used to detect the expression levels of cAMP in vitro model of each group. F (2, 12) = 181.8, *P < 0.0001.* D) Elisa was used to detect the expression levels of cAMP in hippocampus of each group. F (2, 12) = 1.318, *P = 0.3038*. E) Transcript levels of PKA-RI, PKA-RII, PKA-C and CREB in the hippocampus of each group. F) Protein expression levels of key molecules of the cAMP signaling axis in vitro model of each group. PKA-RII^S99^/PKA-RII: t = 14.29, df = 4, *P = 0.0001*; PKA-C^T197^/PKA-C: t = 7.537, df = 4, *P = 0.0017*; CREB^S133^/CREB: t = 7.425, df = 4, *P = 0.0018*. The data were analyzed using one-way ANOVA (**B**, **C** and **D**) or Student’s t test (**F**), and all data are expressed as the mean ± standard deviation. ***P < 0.01*, ****P < 0.001* and *****P < 0.0001* represents a statistically significant difference between the two groups. ns, not significant. Each experiment was repeated three times.


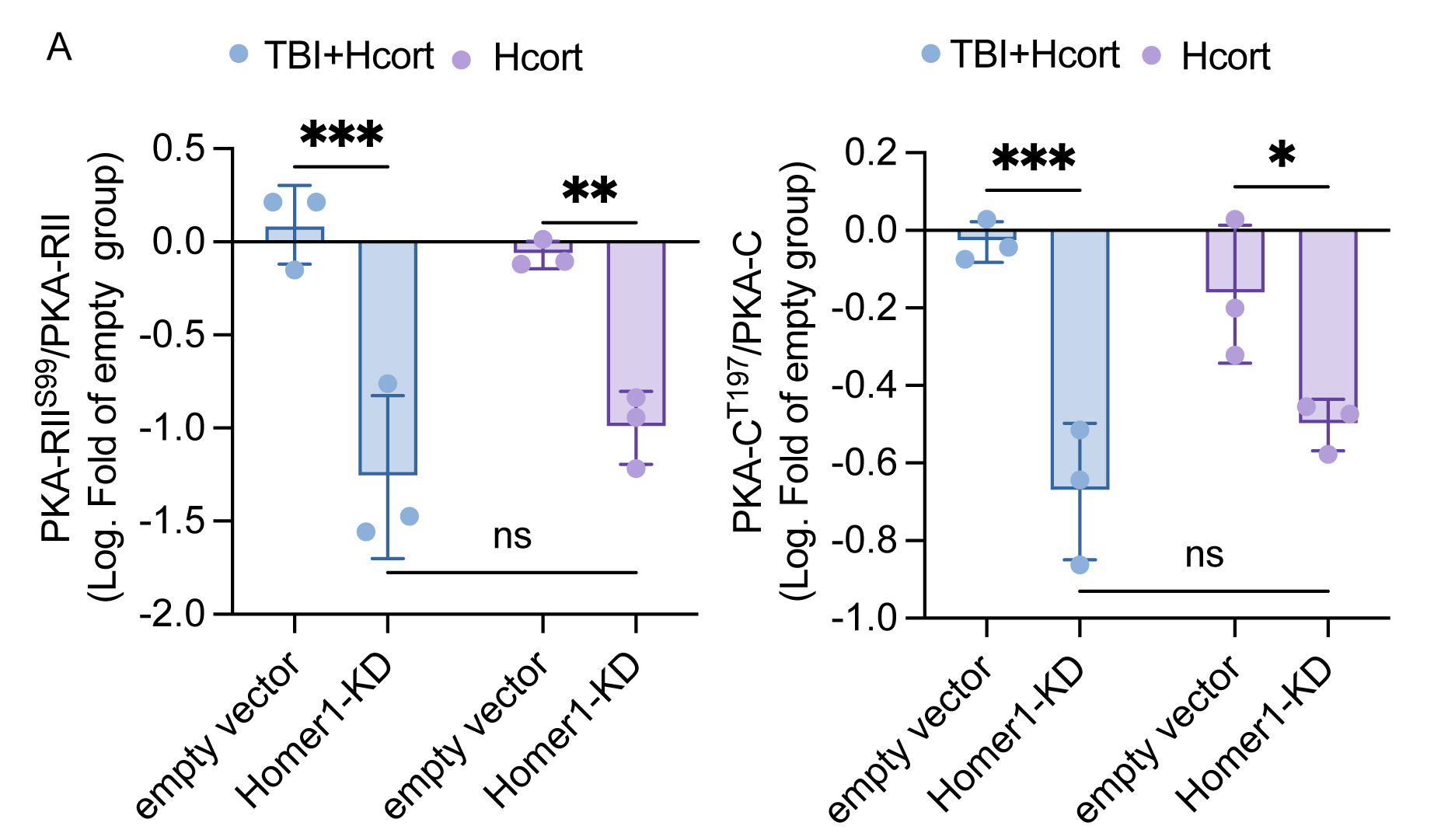


**Figure S7.** Protein expression levels of key molecules of the cAMP signaling axis.

**A:** Quantification of result in Fig. 6C. PKA-RII^S99^/PKA-RII: F_Interaction_ (1, 8) = 1.954, *P = 0.1997*. PKA-C^T197^/PKA-C: F_Interaction_ (1, 8) = 4.042, *P = 0.0792*. The data were analyzed using two-way ANOVA, and all data are expressed as the mean ± standard deviation. **P < 0.05*, ***P < 0.01* and ****P < 0.001* represents a statistically significant difference between the two groups. ns, not significant. Each experiment was repeated three times.


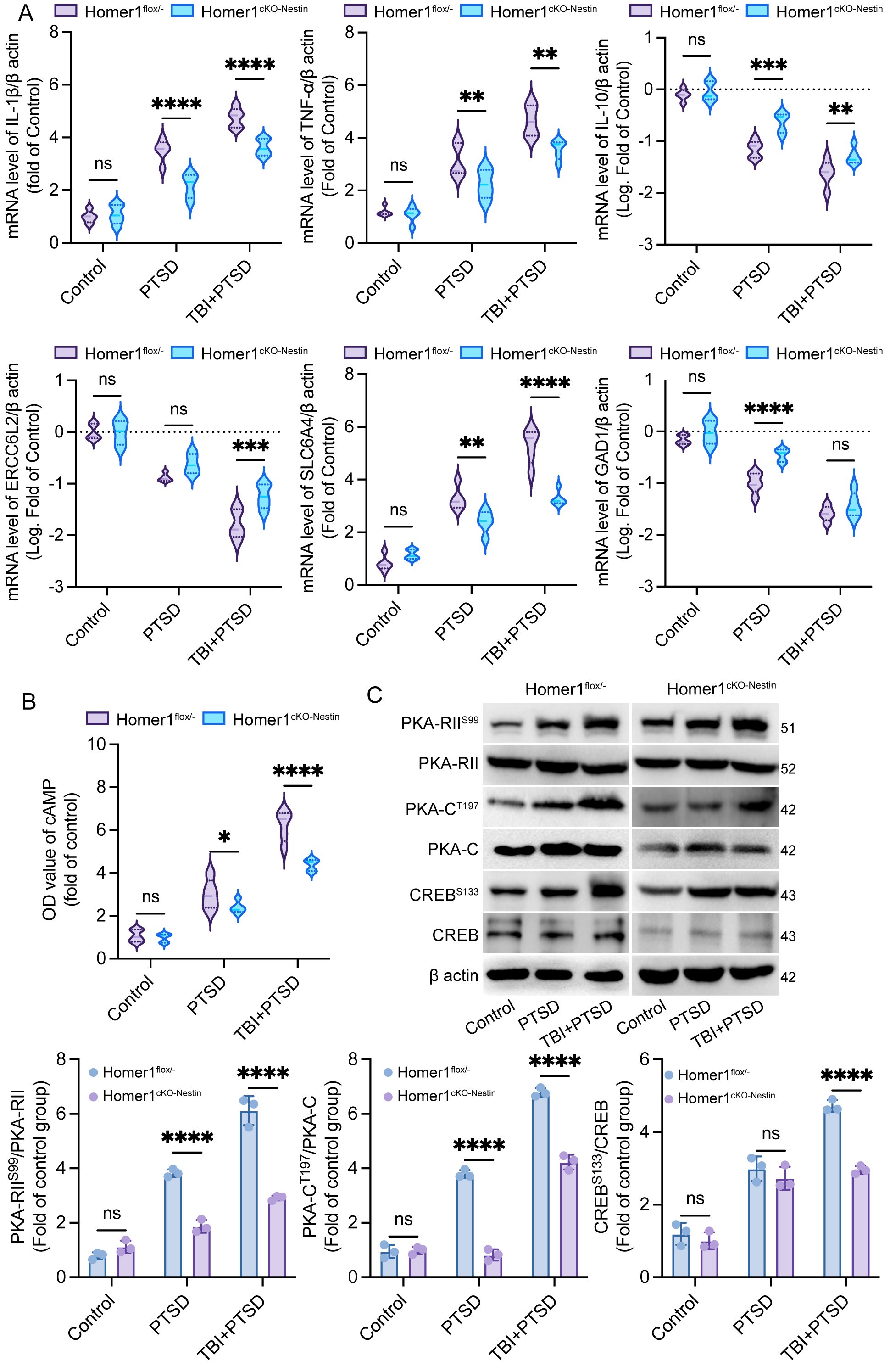


**Figure S8.** Conditional knockout of Homer1 suppresses PTSD formation and cAMP signaling axis.

A) mRNA levels of IL-1β, TNF-α, SLC6A4, IL-10, ERCC6L2, and GAD1 in mouse cortical tissue of Homer1^flox/−^ mice than in Homer1^cKO-Nestin^. IL-1β: F_Interaction_(2, 24) = 10.89, *P = 0.0004*; TNF-α: F_Interaction_(2, 24) = 3.201, *P = 0.0586*; IL-10: F_Interaction_(2, 24) = 3.927, *P = 0.0335*; ERCC6L2: F_Interaction_(2, 24) = 4.370, *P = 0.0241*; SLC6A4: F_Interaction_(2, 24) = 20.90, *P < 0.0001*; GAD1: F_Interaction_(2, 24) = 3.715, *P = 0.0393*; B) Elisa was used to detect the expression levels of cAMP in mouse cortical tissue. F_Interaction_(2, 24) = 9.533, *P = 0.0009*; C) Protein expression levels of key molecules of the cAMP signaling axis in mouse cortical tissue of different groups. PKA-RII^S99^/PKA-RII: F_Interaction_(2, 12) = 66.92, *P < 0.0001*; PKA-C^T197^/PKA-C: F_Interaction_(2, 12) = 99.51, *P < 0.0001*; CREB^S133^/CREB: F_Interaction_(2, 12) = 17.79, *P = 0.0003*; The data were analyzed using two-way ANOVA, and all data are expressed as the mean ± standard deviation. **P < 0.05*, ***P < 0.01*, ****P < 0.001* and *****P < 0.0001* represents a statistically significant difference between the two groups. ns, not significant. Each experiment was repeated three times.


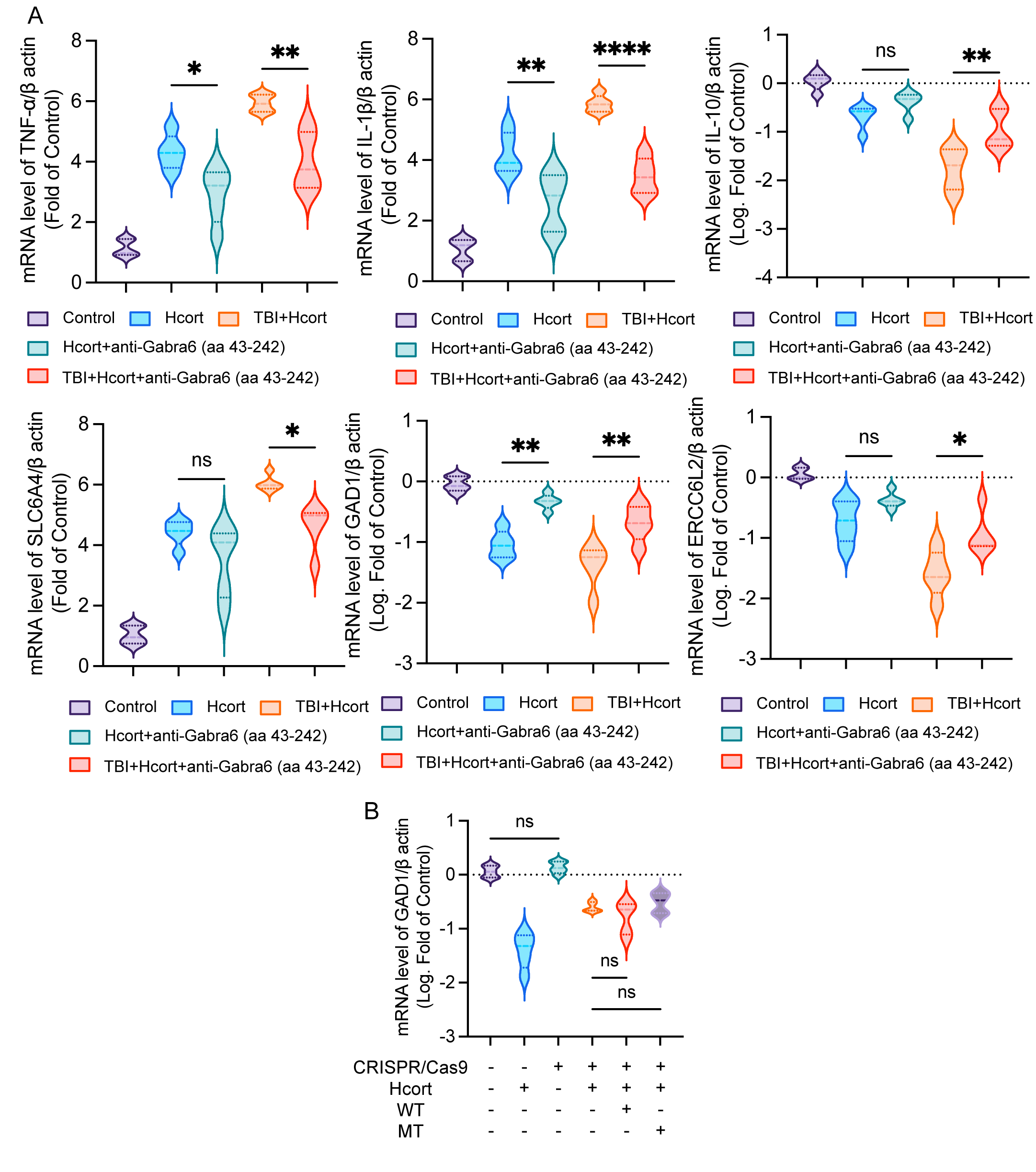


**Figure S9.** the 218th Glu in the Gabra6 sequence may play an important role in inducing the formation of PTSD.

A) mRNA levels of IL-1β, TNF-α, SLC6A4, IL-10, ERCC6L2, and GAD1 after treatment with monoclonal antibodies (2 μg/ml for 24 hours) in vitro model. TNF-α: F (4, 20) = 35.24, *P < 0.0001*; IL-1β: F (4, 20) = 41.44, *P < 0.0001*; IL-10: F (4, 20) = 24.00, *P < 0.0001*; SLC6A4: F (4, 20) = 38.92, *P < 0.0001*; GAD1: F (4, 20) = 23.86, *P < 0.0001*; ERCC6L2: F (4, 20) = 23.65, *P < 0.0001*; B) mRNA levels of GAD1. F (5, 24) = 36.57, *P < 0.0001*; The data were analyzed using one-way ANOVA, and all data are expressed as the mean ± standard deviation. **P < 0.05*, ***P < 0.01* and *****P < 0.0001* represents a statistically significant difference between the two groups. ns, not significant. Each experiment was repeated three times.

**Figure S10.** The causal relationship between PTSD and cerebral cortical structures.

A) Flow chart depicting the Mendelian randomization study investigating the causal relationship between PTSD and brain cortical structure defined by magnetic resonance imaging-measured surficial area and thickness. B) IVW estimates of PTSD effects on brain cortical structure defined by magnetic resonance imaging-measured surficial area and thickness. The color of each block indicates IVW-derived P-values from MR analyses. Significance set at P-values < 0.05/138 after multiple testing correction. SA: Surficial area; TH: thickness.
